# Profilin and Mical combine to impair F-actin assembly and promote disassembly and remodeling

**DOI:** 10.1038/s41467-021-25781-3

**Published:** 2021-09-20

**Authors:** Elena E. Grintsevich, Giasuddin Ahmed, Anush A. Ginosyan, Heng Wu, Shannon K. Rich, Emil Reisler, Jonathan R. Terman

**Affiliations:** 1 Department of Chemistry and Biochemistry, University of California, Los Angeles (UCLA), Los Angeles, CA 90095 USA; 2 Department of Chemistry and Biochemistry, California State University, Long Beach (CSULB), Long Beach, CA 90840 USA; 3 Departments of Neuroscience and Pharmacology, The University of Texas Southwestern Medical Center, Dallas, TX 75390 USA; 4 Molecular Biology Institute, UCLA, Los Angeles, CA 90095 USA

**Keywords:** Drosophila, Cell signalling, Actin, Axon and dendritic guidance

## Abstract

Cellular events require the spatiotemporal interplay between actin assembly and actin disassembly. Yet, how different factors promote the integration of these two opposing processes is unclear. In particular, cellular monomeric (G)-actin is complexed with profilin, which inhibits spontaneous actin nucleation but fuels actin filament (F-actin) assembly by elongation-promoting factors (formins, Ena/VASP). In contrast, site-specific F-actin oxidation by Mical promotes F-actin disassembly and release of polymerization-impaired Mical-oxidized (Mox)-G-actin. Here we find that these two opposing processes connect with one another to orchestrate actin/cellular remodeling. Specifically, we find that profilin binds Mox-G-actin, yet these complexes do not fuel elongation factors’-mediated F-actin assembly, but instead inhibit polymerization and promote further Mox-F-actin disassembly. Using *Drosophila* as a model system, we show that similar profilin–Mical connections occur in vivo – where they underlie F-actin/cellular remodeling that accompanies Semaphorin–Plexin cellular/axon repulsion. Thus, profilin and Mical combine to impair F-actin assembly and promote F-actin disassembly, while concomitantly facilitating cellular remodeling and plasticity.

## Abstract (web summary)

Actin-based structures in cells and tissues are built and maintained through a poorly understood balance between assembly and disassembly. Here, our findings provide insights into how factors known to promote these opposing effects dynamically integrate to shape cells and tissue systems.

## Introduction

**Fig. 1:**
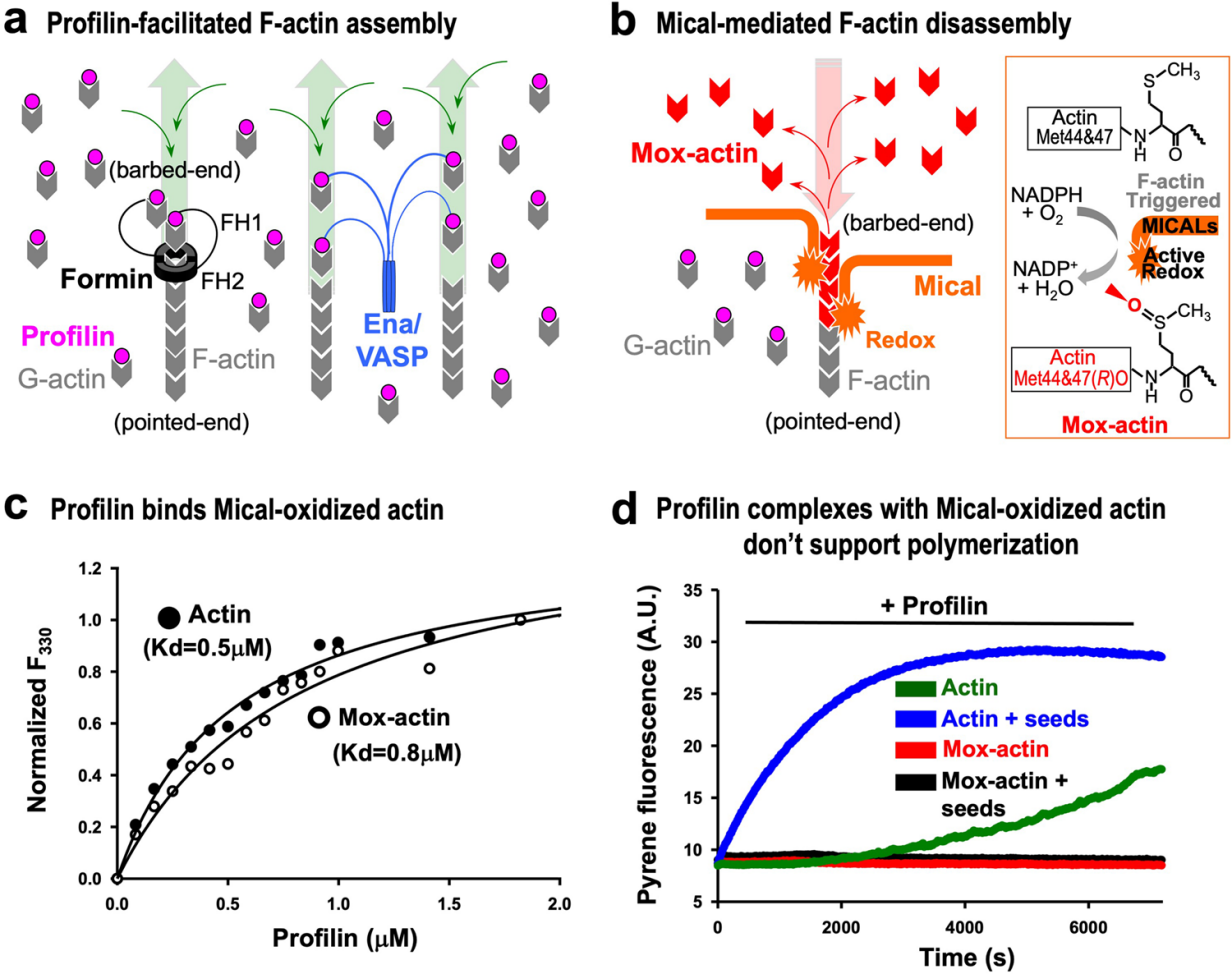
Mical-oxidized actin binds and inhibits profilin-assisted actin polymerization. **a** Profilin facilitates polymerization by fueling F-actin assembly by elongation-promoting factors (formins, Ena/VASP). In particular, most cellular monomeric/G-actin is complexed with G-actin binding proteins such as profilin (pink). These profilin–actin complexes associate with elongation-promoting factors such as formins (black) and Ena/VASP (blue) to drive monomers’ incorporation (thin green arrows) leading to F-actin elongation (thick green arrows) in a tightly controlled manner. Based on current models, formins trigger actin nucleation by stabilizing transient actin dimers. Formins are shown here as dimers, such that each FH1 domain binds profilin–actin and delivers it to FH2 domains, which are bound to actin’s barbed-end. For simplicity, the cell membrane is neither illustrated, nor formins’ or Ena/VASP’s association with it. **b** Mical post-translationally and specifically oxidizes actin to induce F-actin disassembly. In particular, based on current models, Mical with its Redox region (orange, left model and inset) directly associates with F-actin. F-actin triggers Mical’s Redox enzymatic activity (inset)—such that with its co-enzyme NADPH, Mical stereospecifically (in the *R*-isomer conformation) oxidizes Met44 and Met47 residues (inset) in the D-loop at actin’s pointed end. This generates Mical-oxidized actin (Mox-actin) (red, model and inset). Since this oxidation occurs on residues along the interface of actin filament subunits, it weakens the interactions between individual filament subunits and promotes F-actin disassembly (thin red arrows) and shortening (thick red arrow). **c** Profilin binds to Mical-oxidized actin to a similar extent as to unoxidized actin. Binding of human profilin-1 to actin was measured by changes in tryptophan fluorescence of actin. Unoxidized actin (closed circles). Mox-actin (open circles). *n* = 2 (two different preps of both actins) with each data point an average of two independent experiments. Dissociation constants (Kd) for unoxidized and Mox-actin under G-buffer conditions are estimated as 0.5 and 0.8 µM, respectively. [Actin] = 0.15 µM; excitation wavelength (295 nm), emission (330 nm). F_330_ = normalized fluorescence at 330 nm. **d** Profilin complexes with unoxidized actin (blue, green traces), but not with Mical-oxidized actin (red, black traces), polymerize (with or without F-actin seeds). [Actin] = 3 µM; [profilin] = 9 µM; [F-actin-phalloidin seeds] = 0.25 µM (stabilized with phalloidin (Ph) at 1:50 Ph:actin molar ratio). A.U. arbitrary units. *n* = 2 separate experiments with similar results. Source data for Fig. 1 are provided as a Source Data file.

Understanding the factors that regulate the actin cytoskeleton, and the interplay among them, is a critical biomedical goal. Actin reversibly transitions between its monomeric form (G-actin) and double stranded helical polymers (filaments or F-actin), and such dynamic change drives a broad range of cellular processes^1^. In vitro, work with purified monomeric actin is often done in the absence of binding proteins, where actin can be induced to polymerize in the presence of physiologically-relevant divalent cations such as Mg^2+^ ^1^. In vivo, however, the monomeric actin pool is mostly complexed with sequestering proteins—thymosins and profilins (Fig. 1a^2^). Complex formation between G-actin and profilin is especially important physiologically such that this process inhibits spontaneous nucleation of actin, restricting it to tightly controlled nucleation by specialized regulatory proteins^3,4^. Additionally, actin–profilin complexes fuel processive assembly/elongation of actin filaments by elongation-promoting factors such as formins and Ena/VASP (Fig. 1a^3,4^). Specifically, these elongation factors contain polyproline tracks in their sequence that bind profilin, thereby increasing local concentrations of profilin–actin complexes near the barbed ends of filaments and promoting their elongation^3,4^. Therefore, the regulation of profilin levels and its binding to different actin forms is critical for modulation of cellular functions.

In addition to this regulation of actin assembly, cellular structure and organism behavior are also driven by the tightly controlled disassembly of actin filaments. Recently, MICAL family enzymes (which include one *Drosophila* Mical and three mammalian MICALs, MICAL-1, MICAL-2, and MICAL-3) have emerged as important F-actin disassembly proteins in vitro and in vivo (Fig. 1b; reviewed in refs. ^5–8^). MICALs function by employing their flavoprotein monooxygenase (Redox) domain to carry out the site-specific post-translational modification (stereospecific oxidation) of two amino acid residues in actin’s DNaseI-binding loop (methionine (Met)44 and Met47), which is a part of F-actin’s self-assembly interface (Fig. 1b, inset^9–12^). MICALs preferentially affect actin that is in the filamentous state (F-actin)—and F-actin, but not monomeric (G-) actin, strongly triggers MICALs’ enzymatic activity and oxidation of actin (Fig. 1b, inset^9,10,13^). MICALs-induced oxidation of actin also affects F-actin stability in a nucleotide-state sensitive manner, exhibiting stronger destabilizing effects on ADP-bound MICALs-oxidized (Mox)-F-actin with disassembly rates of >80 actin subunits/s^11^. Also, while the critical concentration of ATP-bound Mox-actin is at least one order of magnitude higher (~1 µM) than that of unoxidized actin^14^, ADP-bound Mox-actin monomers do not appear to polymerize even at high concentrations (>30 µM)^11^. In addition, when present in conjunction with the ubiquitous F-actin disassembly protein cofilin, oxidation of actin by MICALs promotes rapid F-actin severing and depolymerization even in the presence of inorganic phosphate^14,15^. The MICALs therefore disassemble F-actin and release oxidized actin monomers into the G-actin pool (Fig. 1b). Yet, it is unknown whether Mox-G-actin monomers can associate with monomer binding proteins like profilin. It is also unknown, whether Mox-actin is reincorporated into actin-based structures by profilin-dependent elongation-promoting factors—thereby resulting in compounded, long-range detrimental effects of MICALs-mediated actin oxidation on F-actin stability.

Here, we show that Mical-oxidized actin (Mox-actin) interacts with the actin binding protein, profilin. Yet, we find that profilin–Mical-oxidized actin complexes cannot be used by actin elongation-promoting factors (formins and Ena/VASP) to nucleate/polymerize actin. Further, we find that profilin preferentially destabilizes Mox-actin filaments. Our results also reveal that profilin and Mical combine in vivo to facilitate actin-driven cellular remodeling and axon guidance. Our results, therefore, indicate that profilin and Mical join together to trigger a switch from a cellular program promoting F-actin assembly to one impairing assembly and driving F-actin disassembly and remodeling.

## Results

### Profilin binds Mical-oxidized actin but these complexes do not support actin polymerization

MICALs-mediated post-translational oxidation of actin has now emerged as a critical regulator of actin dynamics (reviewed in refs. ^5–8^), yet how MICALs may interplay with actin assembly proteins is unknown. We therefore sought to determine how oxidation of actin by Mical may affect actin’s regulation by other proteins. Profilin is the best-known G-actin sequestering protein and a critical controller of actin polymerization. To test how profilin interacts with Mical-oxidized (Mox)-actin we used an established assay that is based on the quenching of the intrinsic fluorescence of actin upon its binding to profilin^16^. Interestingly, we observed similar profilin binding to Mox- and unoxidized actin (Fig. 1c). These results indicate that profilin has the ability to associate with Mox-actin and is also consistent with our previous observations that profilin accelerates nucleotide exchange in unoxidized and Mox-actin to the same extent^11^.

Since we found that profilin could bind Mox-actin, we next wondered if this actin could polymerize in the presence of profilin. Markedly, we observed striking differences between profilin’s effect on the polymerization of unoxidized actin versus Mox-actin. In particular, in the presence of profilin, unoxidized actin polymerizes slowly but Mox-actin does not polymerize at all (Fig. 1d, compare traces of Mox- and unoxidized actin; and see below that Mox-actin in the presence of profilin does not polymerize even after overnight (24 h) incubation). Furthermore, since profilin is known to inhibit actin nucleation, we introduced unoxidized actin seeds into the reactions to bypass the nucleation step and monitor the effect of profilin on elongation of Mox- and unoxidized actin (Fig. 1d). Notably, actin that had been oxidized by Mical could not be induced to polymerize in the presence of profilin even when unoxidized F-actin seeds were added (Fig. 1d). Thus, our results indicate that unlike unoxidized actin, profilin complexes with Mical-oxidized actin do not polymerize.

### Profilin–Mox-actin complexes do not support actin polymerization mediated by formins and Ena/VASP

**Fig. 2:**
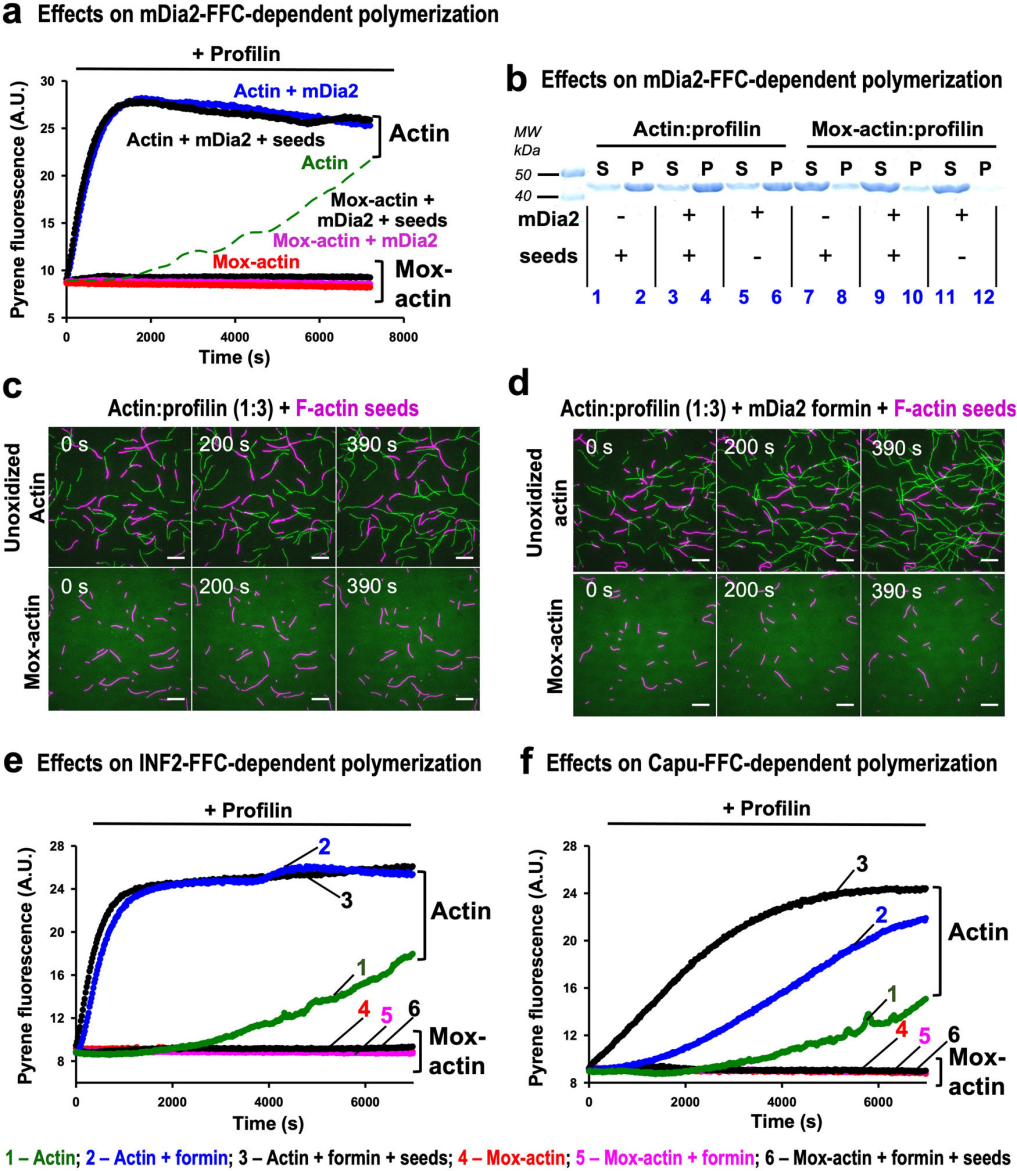
Mox-actin–profilin complexes markedly inhibit formin-dependent actin polymerization. **a**–**d** Assembly of unoxidized and Mox-actin in the presence of profilin and mDia2-FFC formin construct (with or without phalloidin-stabilized F-actin seeds). Results obtained by three different methods—pyrene fluorescence measurements (**a**), high-speed co-sedimentation (**b**), and TIRF microscopy (**c**, **d**)—are consistent with the absence of mDia2-FFC- -driven Mox-actin assembly in the presence of profilin. *n* = 2 separate experiments with similar results for each of **a**–**d**. **a**, **b** [Actin] =3 µM; [profilin] = 9 µM; [mDia2-FFC] = 30 nM; [F-actin-phalloidin seeds] = 0.25 µM (stabilized with phalloidin (Ph) at 1:50 Ph:actin molar ratio). A.U. arbitrary units. **c**, **d** [Actin] = 0.5 µM, [Mox-actin] = 1.4 µM (0.4 µM above each of their critical concentrations); Profilin-to-actin ratios = 3:1. [mDia2-FFC] = 1.5 nM. Note that the profilin–actin control sample in (**a** green trace) is a smoothed fit through the data points and is therefore shown as a dashed line (see Source Data file). This condition is repeated in the green traces in Figs. 1d, 2e, 2f, and Supplemental Fig. 2. Scale bars (**c**, **d**) = 10 µm. **e**, **f** INF2 (**e**) and *Drosophila* Capu (**f**) formins do not support Mox-actin assembly in the presence of profilin. Traces obtained are labeled as follows (similar to Fig. 2a): 1—Actin; 2—Actin + formin; 3—Actin + formin + seeds; 4—Mox-actin; 5—Mox-actin + formin; 6—Mox-actin + formin + seeds. *n* = 2 separate experiments with similar results for each of **e** and **f**. [Actin] = 3 µM; [profilin] = 9 µM; [INF2-FFC] = 10 nM; [Capu-FFC] = 20 nM; [F-actin-phalloidin seeds] = 0.25 µM (stabilized with phalloidin (Ph) at 1:50 Ph:actin molar ratio). Note that formins have different potencies in promoting actin assembly (e.g., refs. ^78,79^). Therefore, different concentrations of formins’ were used in the reactions. Source data for Fig. 2 including uncropped gels are provided as a Source Data file.

Since actin–profilin complexes couple with nucleating/elongation-promoting factors such as formins to drive processive elongation of actin filaments, we wondered if Mox-actin–profilin complexes could be utilized by actin nucleating/elongation-promoting factors. To begin examining the effects of actin nucleating/elongation-promoting factors on assembly of Mox-actin, we employed the well-known/ubiquitous formin mDia2^3,4^. Notably, in the presence of mDia2-FFC (a nonautoinhibited form of mDia2 was used), unoxidized actin–profilin complexes rapidly polymerized (Fig. 2a, blue trace), but Mox-actin–profilin complexes did not polymerize (Fig. 2a, magenta trace). Furthermore, to bypass the nucleation step, we supplemented the mDia2-FFC reactions with unoxidized, phalloidin-stabilized F-actin seeds (Fig. 2a, black traces). Markedly, no polymerization of Mox-actin was observed even under these conditions (Fig. 2a, flat black trace). We also confirmed these observations using high-speed sedimentation assays. As expected, in the presence of profilin and formin, unoxidized actin was recovered in the pellet (F-actin) (Fig. 2b, lanes 4 and 6). In contrast, under the same conditions, Mox-actin was recovered in the supernatant (as G-actin) even in the presence of unoxidized F-actin seeds (Fig. 2b, lanes 9 and 11). We also monitored this system directly by employing TIRF microscopy (Fig. 2c-d). We deposited phalloidin-stabilized unoxidized F-actin seeds (Fig. 2c-d, magenta colored) on the slide surface and monitored their elongation with profilin–actin complexes in the absence (Fig. 2c) and presence of mDia2-FFC (Fig. 2d). Upper panels in Fig. 2c-d showed that profilin, in complex with unoxidized actin, supports filaments’ growth (in green). In contrast, we observed no elongation from the F-actin seeds in the presence of Mox-actin–profilin complexes with or without mDia2-FFC (Fig. 2c-d, lower panels). Moreover, we found that SelR/MsrB, which is an enzyme that reduces Mox-actin^12,17^, reverses Mox-actin’s effect on polymerization by profilin/mDia2-FFC and enables polymerization again (Supplementary Fig. 1). Thus, our results indicate that actin that has been oxidized by Mical hinders profilin-facilitated actin polymerization by formin.

We next wondered whether Mox-actin–profilin’s effects were specific to the mDia2 formin or were similar with other actin elongators. To this end we employed INF2 and *Drosophila* cappuccino (Capu), which belong to formin classes other than diaphanous (Dia)^18^. As in the experiments with mDia2, we used FFC constructs of these proteins (containing FH1, FH2 domains, and tail region) to bypass formins’ autoinhibition. We found that similar to mDia2-FFC, these actin elongation-promoting factors did not support the polymerization of Mox-actin–profilin complexes, even in the presence of unoxidized F-actin seeds (Fig. 2e-f). Moreover, we also tested enabled (Ena/VASP), a well known and potent elongator of actin bundles^19^. Similar to our results with the different classes of formins, we found that Ena/VASP did not support the polymerization of Mox-actin–profilin complexes, even in the presence of unoxidized F-actin seeds (Supplementary Fig. 2). Thus, Mox-actin–profilin complexes inhibit the actin polymerization activity of formin and Ena/VASP actin elongation-promoting factors.

### Mox-actin–profilin, similar to unmodified actin–profilin complexes, interacts with the polyproline tracks of formins

We next sought to gain mechanistic insight into the observed inhibition of formin-mediated actin assembly in the presence of Mox-actin and profilin. The FH1 domain of formins is an actin-recruiting region where actin–profilin complexes bind to polyproline tracks in the FH1 domain of formins. In general, the affinity of profilin to FH1 domain polyproline stretches is low (tens of micromolar range)^3^, but it has been reported that binding to actin increases the affinity of profilin for these polyproline tracks by up to an order of magnitude^20^. Therefore, it is feasible that profilin complexes with unconventional/post-translationally modified actins (such as Mox-actin) may have greatly reduced affinities to these polyproline tracks and thereby not support formin-mediated processive elongation. Thus, we carried out pull-down experiments with a GST-tagged mDia2FH1-FH2 formin construct (mDia2-FF) to probe for any differences in the amounts of bound actin (Mox- and unoxidized) and profilin (Supplementary Fig. 3). We used conditions under which most of the actin is complexed with profilin and, therefore, would not be expected to interact with the mDia2-FH2 domain—the region that binds uncomplexed actin. We chose to employ the mDia2-FF construct because it has a truncated tail region, which weakens further its interaction with any free (uncomplexed) actin. In these experiments, we used 0.5, 5, and 15 µM of GST-mDia2-FF dimer (with 4 polyproline tracks), actin, and profilin, respectively. After accounting for nonspecific binding and formin amounts, we obtained the following binding stoichiometries (per 1 formin dimer): for unoxidized actin 0.81 ± 0.18 and 1.03 ± 0.33 of actin and profilin, respectively (*n* = 3 pull-down experiments) and for Mox-actin 0.62 ± 0.13 and 0.58 ± 0.13 of actin and profilin, respectively (*n* = 3 pull-down experiments). These results show that Mox-actin–profilin complexes bind to polyproline tracks of mDia2 and that this interaction, although somewhat decreased, is not dramatically affected by Mical-induced oxidation of actin.

### Mical-mediated oxidation of actin does not preclude its interaction with formins

**Fig. 3:**
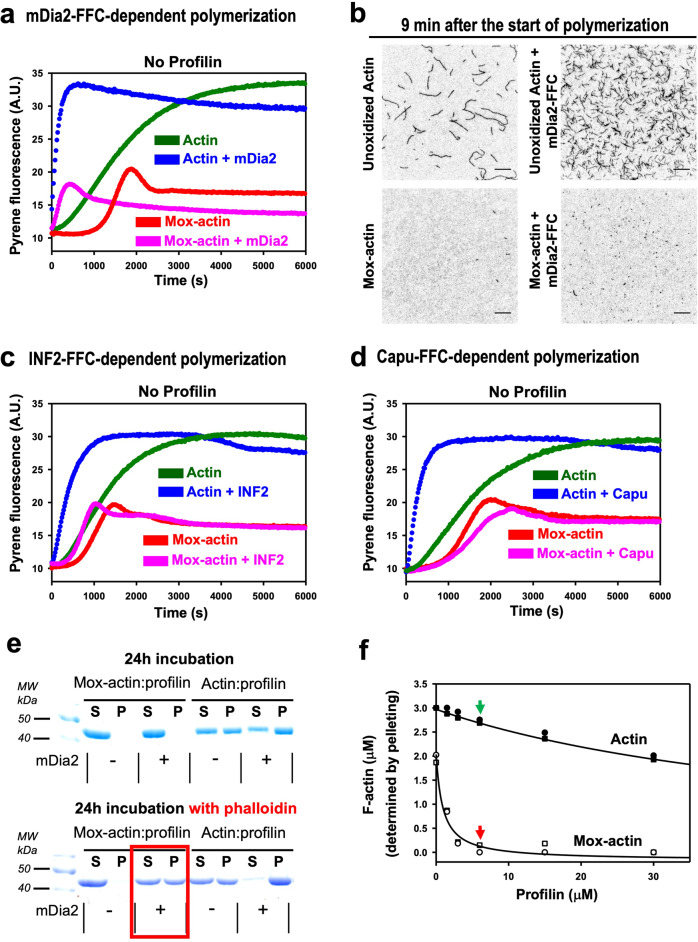
Mox-actin and profilin combine to inhibit formin-dependent actin polymerization and further promote F-actin disassembly. **a**, **b** mDia2-FFC nucleates both unoxidized and Mox-F-actin formation. **a** Pyrene fluorescence assays. In the presence of mDia2-FFC (and without profilin) both unoxidized and Mox-actin polymerize without a lag phase. mDia2-FFC or other formins (**c**, **d**) do not increase the extent of polymerization. *n* = 3 separate experiments with similar results. [Actin] = 3 µM; [mDia2-FFC] = 30 nM. The lower Mox-actin plateau level/fluorescence (in **a**, **c**, **d**) compared to unoxidized actin’s higher plateau level/fluorescence correlates with Mox-actin’s increased critical concentration relative to unoxidized actin^10–12,14^. A.U. arbitrary units. **b** TIRF microscopy. mDia2-FFC induces formation of larger numbers of filaments in unoxidized and Mox-actin samples. Mox-actin filaments appear very short. *n* = 2 samples, 6–7 random fields were imaged/condition with similar results. [Actins] = 3 µM and [mDia2-FFC] = 30 nM both diluted 150 fold (see Methods for details). Scale bars = 10 µm. **c**, **d** Pyrene fluorescence assays. **c** Mox-actin’s polymerization lag phase is shortened in the presence of INF2-FFC formin (and without profilin). *n* = 3 separate experiments with similar results. [Actin] = 3 µM; [INF2-FFC] = 10 nM. **d** Capu-FFC does not aid Mox-actin polymerization even without profilin. *n* = 3 separate experiments with similar results. [Actin] = 3 µM; [Capu-FFC] = 20 nM. **e**, **f** Profilin preferentially destabilizes Mox-F-actin. (**e**) Prolonged incubation of Mox-actin–profilin complexes with mDia2-FFC and phalloidin (under polymerizing conditions) results in filaments formation. Top: with profilin, no Mox-actin polymerization was observed with mDia2-FFC in high-speed pellets even after 24 h incubation. Lower: Mox-actin presence in high-speed pellets (after incubation with profilin, mDia2-FFC, and phalloidin) indicates phalloidin-induced stabilization of transiently-formed Mox-actin filaments nucleated by mDia2 (red frame). *n* = 2 separate experiments with similar results. [Actin] = 3 µM; [profilin] = 9 µM; [mDia2-FFC] = 30 nM; [phalloidin] = 3.3 µM. Note that since ATP hydrolysis could take place over 24 h incubation, phalloidin was added at the beginning of polymerization to capture any transiently-formed filaments. This was necessary because ADP-bound Mox-actin does not polymerize even at high concentrations^11^. **f** Increasing profilin strongly decreases levels of Mox-F-actin. [G-Actin] of 3 µM; KMEH, pH 7 was used to assemble F-actin. Actin amounts in resuspended pellets (F-actin) determined by densitometry analysis (SDS-PAGE/Coomassie staining). Two independent experiments shown. Closed symbols (actin), open symbols (Mox-actin). Arrows (green, red) demarcate results from 1:2 molar ratio of actin:profilin. Source data for Fig. 3 including uncropped gels are provided as a Source Data file.

We wondered if Mox-actin’s inhibitory effects on formin-driven actin polymerization may be mediated through profilin. Based on the current model, formins trigger actin nucleation by stabilizing transient actin dimers (illustrated in Fig. 1a^3,4^). Structurally, the dimerized FH2 domain of formins is necessary and sufficient for actin nucleation and barbed-end binding. Additionally, the tail region of formins aids these activities (Fig. 1a^21^). We therefore considered a possibility that the Mical-mediated oxidation of actin’s Met44/Met47 residues may weaken the interaction between actin monomers and the FH2-tail region of formins, and thereby abolish formins’ ability to interact with and nucleate actin. To test this idea, we assessed the effects of formins on Mox-actin in the absence of profilin. Notably, we found that in the absence of profilin, Mox-actin is polymerized by formins—and this occurs to different extents for different formins (Fig. 3). Specifically, we have previously found that at high enough concentrations, Mox-actin polymerizes, but it exhibits a long lag-time in polymerization due to a delay in nucleation (Fig. 3a, red trace^14^). In contrast, the addition of the mDia2-FFC formin protein eliminates the lag phase of Mox-actin polymerization, indicating that the mDia formin directly interacts with Mox-actin and aids its nucleation (Fig. 3a, compare red and magenta traces). We confirmed this result in independent experiments employing TIRF microscopy (Fig. 3b). Imaging of the diluted reaction mixtures, immobilized on a polylysine surface, showed that upon addition of mDia2-FFC, more of the short Mox-actin filaments were present on the surface compared to those of Mox-actin alone (Fig. 3b, compare the lower right and left panels). We also observed shortening of the lag phase of actin assembly with IFN2-FFC (Fig. 3c, compare red and magenta traces), but not to the extent seen with mDia2 (Fig. 3a). In contrast, the duration of the lag phase observed in Mox-actin polymerization was not shortened by Capu-FFC (Fig. 3d, compare red and magenta traces)—and indeed we noticed that the Capu formin moderately slowed down the overall kinetics of Mox-actin polymerization compared to Mox-actin alone (Fig. 3d). Thus, these results indicate that Mox-actin can associate with formins, and in the absence of profilin, some formins (mDia2 and IFN2) can utilize Mox-actin and enhance its assembly rate. It is also notable that nucleation potencies of these three formins towards Mox-actin mirror the trend previously observed with unoxidized actin^21–23^, where the diaphanous (Dia) formins are known to be the most potent actin nucleators.

### Mox-actin filaments are destabilized by profilin to a greater extent than unoxidized F-actin

Our results indicate that profilin interacts with Mox-actin but their complex does not contribute to actin filament nucleation/elongation. Moreover, we find that profilin and its complex with Mox-actin interacts with formins, but formins and Ena/VASP cannot mediate the incorporation of such complexes into filaments. Thus, our results support the view that profilin in combination with Mox-actin negatively affects actin polymerization. Along these lines, it is also interesting that profilin is known to increase the depolymerization rate of unoxidized F-actin^24–26^ —and such destabilizing effects are observed even in the presence of formins^27–29^. Considering the unique structure and dynamic properties of Mox-F-actin, we wondered if its filaments/oligomers could be destabilized by low concentrations of free profilin used in standard assays. Furthermore, since mDia2 aids in nucleation of uncomplexed Mox-actin (e.g., compare Fig. 3a with Fig. 2a and Fig. 1d), and also interacts with Mox-actin when it is in complex with profilin (Supplementary Fig. 3), we hypothesized that the formin-induced assembly of Mox-actin filaments counterbalances their destabilization by free profilin. To test this idea, we employed a prolonged incubation of mixtures containing Mox-actin, profilin, and mDia2-FFC. Furthermore, we reasoned that under such conditions phalloidin might capture formin-polymerized Mox-actin filaments/oligomers, protecting them from profilin-mediated disassembly. Our results revealed that in the presence of profilin, Mox-actin does not polymerize even after overnight (24 h) incubation (Fig. 3e, top panel). However, we found that when mDia2-FFC and phalloidin were both added into reactions, Mox-actin was detected in high-speed pellets (i.e., in filaments) even in the presence of profilin (Fig. 3e, lower panel, red frame). These results indicate that free profilin disassembles Mox-actin filaments formed by formins.

To further test the effects of profilin on the stability of Mox-actin filaments, we incubated aged F-actin with increasing concentrations of profilin for two hours and used high-speed centrifugation to evaluate the amounts of polymer left in the solution. We observed a prominent destabilization of Mox-actin filaments by profilin (compared to unoxidized actin) (Fig. 3f). For example, at a 1:2 actin:profilin ratio, while ~90% of unoxidized actin remained filamentous (green arrow in Fig. 3f), virtually all Mox-actin was found in the high-speed supernatant (i.e., depolymerized) (red arrow in Fig. 3f). Likewise, decreasing the amounts of profilin in the reaction resulted in an increase in the amount of filamentous Mox-actin (Fig. 3f, open symbols). For instance, close to 30% of filamentous actin was detected at 2:1 ratio of Mox-actin:profilin. At this ratio, based on the Kd estimate (0.8 µM) (Fig. 1c), the amount of profilin–Mox-actin formed in this system would be ~1.1 µM (out of 3 µM total) leaving ~1.9 µM of Mox-actin free. Under such conditions, the concentration of free Mox-actin (uncomplexed with profilin) is above its critical concentration (~1 µM) and, therefore, some filaments should be—and are —present in the reaction (Fig. 3f). In total, therefore, our results with purified proteins indicate that: (i) profilin binding to Mox-actin inhibits profilin-assisted actin polymerization, and (ii) profilin preferentially destabilizes Mox-actin filaments.

### Mical and profilin functionally interact in vivo to induce F-actin and cellular remodeling

**Fig. 4:**
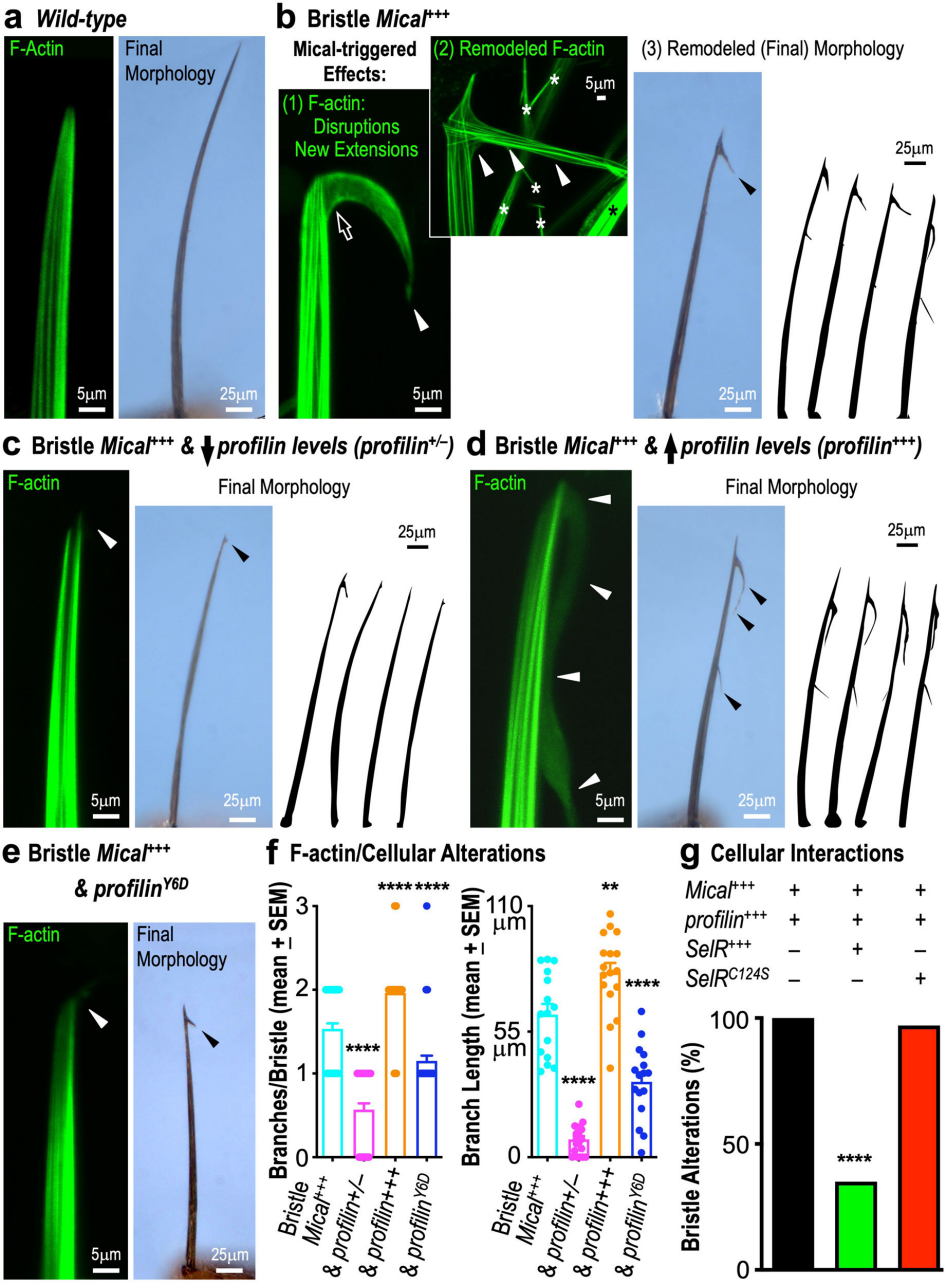
Mical and profilin functionally interact in vivo to direct F-actin and cellular remodeling. F-actin visualized with *UAS:*^*GFP*^*actin/+*. **a**, **b** Mical-mediated F-actin disassembly and cellular remodeling in vivo. **a** The F-actin rich single-cell bristle process is typically unbranched. **b** Increasing *Mical* levels in bristle cells (Bristle *Mical*^*+++*^ = *UAS:Mical/+, B11-GAL4/+*) results in Mical-triggered effects that initially induce F-actin disruptions at the bristle tip ((1), open arrow). F-actin then reassembles to drive bristle extension in a new direction ((1), white arrowhead), which increases over time ((2), white arrowheads)—generating a final branched bristle morphology ((3), black arrowhead, drawings). Asterisks (adjacent bristles/extensions in field of view). **c** Decreasing *profilin* levels in the Bristle *Mical*^*+++*^ background (*UAS:Mical/+, B11-GAL4/+, chic*^*1320*^/+) decreases Mical-triggered F-actin reorganization and cellular remodeling (white and black arrowheads, drawings). **d** Increasing *profilin* levels in the Bristle *Mical*^*+++*^ background (*UAS:Mical/+, B11-GAL4/+, UAS:profilin (1M)/+*) increases Mical-mediated F-actin reorganization and cellular remodeling (white and black arrowheads, drawings). *UAS:profilin* had no effects on its own (*B11-GAL4/+, UAS:profilin (1M)/+*) in these conditions. **e** Mutating profilin’s ability to support actin assembly (profilin^Y6D^) in the Bristle *Mical*^*+++*^ background (*UAS:Mical/+, B11-GAL4/+, UAS:profilin*^*Y6D*^ *(4F)/+*), decreases Mical-mediated F-actin/cellular remodeling (white and black arrowheads). Note, wild-type profilin is still present, so F-actin levels appear relatively normal but Mical’s effects on F-actin remodeling are suppressed. *UAS:profilin*^*Y6D*^ had no noticeable effects on its own (*B11-GAL4/+, UAS: profilin*^*Y6D*^ *(4F)/+*) in these conditions. **f** Quantifying branch number per bristle (left) and branch length (right) from **b**–**e**. Bristle *Mical*^*+++*^ (Branches/Bristle: *n* = 60 cells assessed across 30 animals; Length: *n* = 15 cells assessed across 15 animals). and profilin^+/–^ (Branches/Bristle: *n* = 44 cells assessed across 22 animals; Length: *n* = 20 cells assessed across 20 animals). and profilin^+++^ (Branches/Bristle: *n* = 56 bristle cells assessed across 28 animals; Length: *n* = 17 cells assessed across 17 animals). and profilin^Y6D^ (Branches/Bristle: *n* = 46 bristle cells assessed across 23 animals; Length: *n* = 16 cells assessed across 16 animals). ****p < 0.0001, **p = 0.0067; unpaired *t*-test (two-*t*ailed). **g** Active SelR (SelR^+++^), but not an enzyme-dead version of SelR (SelR^C124S^)^12^, significantly reverses Mical and profilin’s combined effects on F-actin reorganization and cellular remodeling. Genotypes as in **d** (*n* = 72 bristle cells assessed across 36 animals), but also with *UAS:SelR/+* (*n* = 80 bristle cells assessed across 40 animals) or *UAS:SelR*^*C124S*^*/+* (*n* = 68 bristle cells assessed across 34 animals). ****p < 0.0001; *Χ*^2^ test. ≥2 independent experiments were performed with similar results for each of (**a**–**g**). Source data for Fig. 4 are provided as a Source Data file.

In light of our observations with Mical-oxidized actin and profilin in vitro using purified proteins, we wondered if similar combined effects of Mical and profilin were occurring in vivo. Mical and its Redox-driven actin regulatory system is critical for the formation and function of multiple tissues—playing important roles in different cells including among others, neurons, muscles, immune cells, and cancer cells (reviewed in refs. ^5–8^). Also, among the tissues known to be affected by Mical and its effects on F-actin are *Drosophila* bristle cells, which are neuronal mechanosensory cells that have long served as a model for studying actin dynamics and cellular remodeling in vivo (Fig. 4a, Supplementary Fig. 4a^30,31^). In particular, unlike the slightly curved, unbranched bristles of wild-type flies (Fig. 4a, Supplementary Fig. 4a), Mical is required to regulate actin dynamics to shape bristles (i.e., Mical^−/−^ (knockout) mutants exhibit defects in bristle F-actin organization and morphology; Supplementary Fig. 4b^9,31^), and increasing Mical levels in bristles triggers actin/bristle remodeling (Fig. 4b, Supplementary Fig. 4c-f^9,31^). Interestingly, profilin (also known as chickadee (chic) or stranded (sand) in *Drosophila*) is also required to regulate bristle F-actin organization and morphology (i.e., *profilin*^−/−^ (knockout) mutants exhibit defects in bristle F-actin organization and morphology)^32^—and increasing profilin levels in bristles induces actin/bristle alterations (Supplementary Fig. 4g^33^). We therefore used the bristle model system to look at profilin’s involvement in Mical-triggered effects in vivo.

Similar to the leading edge of motile cells^1^, bristle elongation is dependent on actin assembly (Supplementary Fig. 4a^30^). Profilin is subcellularly located throughout the bristle and promotes actin polymerization at free barbed ends, balancing the activity of capping protein to precisely elongate F-actin and direct bristle extension and morphology^32–34^. In contrast, Mical is preferentially positioned subcellularly at the bristle tip (Supplementary Fig. 4d^9,35,36^)—and uses its Redox activity to both disassemble F-actin at the elongating tip and inhibit continued bristle elongation in the same direction (Fig. 4b (1), Supplementary Fig. 4c, e^9,10,12,14,35,36^). Yet, interestingly, in response to these disruptive effects of Mical on F-actin and continued bristle elongation, new F-actin rich branches are formed at the bristle tip and these branches extend in a new direction (Fig. 4b(1-3), f; Supplementary Figs. 4c, 4e-f, 5a^9,10,12,14,35,36^). We therefore wondered if profilin might be involved in regulating Mical’s effects on F-actin and cellular remodeling. Notably, in the process of performing a large-scale genetic screen to look for enhancers and suppressors of Mical-mediated effects on F-actin^12,36^, we found that decreasing the levels of *profilin* (even just with a *profilin* heterozygous (+/–) mutant) significantly suppressed Mical-mediated F-actin reorganization and bristle branching (Fig. 4c, f; Supplementary Fig. 5b-c). Furthermore, increasing the levels of *profilin* significantly enhanced Mical’s effects on F-actin reorganization and cellular remodeling, generating more and longer branches (Fig. 4d, f). Moreover, we found that active SelR, the enzyme that reverses Mical-mediated oxidation of actin^12,17^, reversed profilin’s ability to enhance Mical-mediated F-actin reorganization/cellular remodeling (Fig. 4g). Thus, Mical, Mox-actin, and profilin are functionally connected in vivo to induce F-actin and cellular remodeling.

The effects of decreasing the levels of *profilin* on Mical effects in vivo (Fig. 4c, Supplementary Fig. 5b) are similar to those with other proteins identified as working with Mical including Plexin, cofilin, Abl, and myosin 15^9,14,35,36^—revealing that profilin is required for Mical’s full effects on F-actin remodeling and new branch formation. Therefore, since our results revealed that profilin was required for this new F-actin assembly-driven branch formation, we wondered if this was occurring through its ability to couple with actin elongation-promoting factors. To test this possibility, we sought to dampen all profilin-dependent actin elongation-promoting factors in bristles and generated flies containing a profilin Y6D mutant (profilin^Y6D^)—a mutation known to induce a lower affinity of profilin for the polyproline tracks of actin elongation-promoting factors^37^. Our results revealed that expression of *profilin*^*Y6D*^ in bristles alone (in a wild-type background (without increasing *Mical*)) gave rise to *profilin*^−/−^ mutant-like (and actin elongation-promoting factor knockdown-like) stunted bristle extension defects (Supplementary Fig. 5d^32–34,38^). These results support previous findings that profilin promotes actin polymerization to precisely elongate F-actin and direct bristle extension and morphology^32–34^. We next sought to determine the effects of the *profilin*^*Y6D*^ mutant on Mical-mediated F-actin/cellular remodeling. In particular, since *profilin*^*−/−*^ mutants rarely survive to adulthood^32^, we designed our experiments not to remove wild-type profilin and replace it with profilin^Y6D^, but to compare profilin^Y6D^’s effects on Mical to what we had seen with wild-type profilin (Fig. 4d, f). Our results revealed that the *profilin*^*Y6D*^ mutant attenuated *profilin*’s ability to increase Mical-mediated F-actin reorganization/cellular remodeling (compare Fig. 4e, f to Fig. 4d, f). Moreover, we found that profilin^Y6D^ worked as a dominant negative protein (i.e., similar to decreasing *profilin* levels), such that in the presence of profilin^Y6D^, Mical generated shorter branches than normal (compare Fig. 4e, f to Fig. 4b, f). Together, our in vivo results, support our results with purified proteins, and indicate that Mical—through its Redox-mediated effects on F-actin—combines with profilin to regulate F-actin organization and cellular remodeling.

### Semaphorin–Plexin–Mical repulsion works with profilin to properly guide axons

**Fig. 5:**
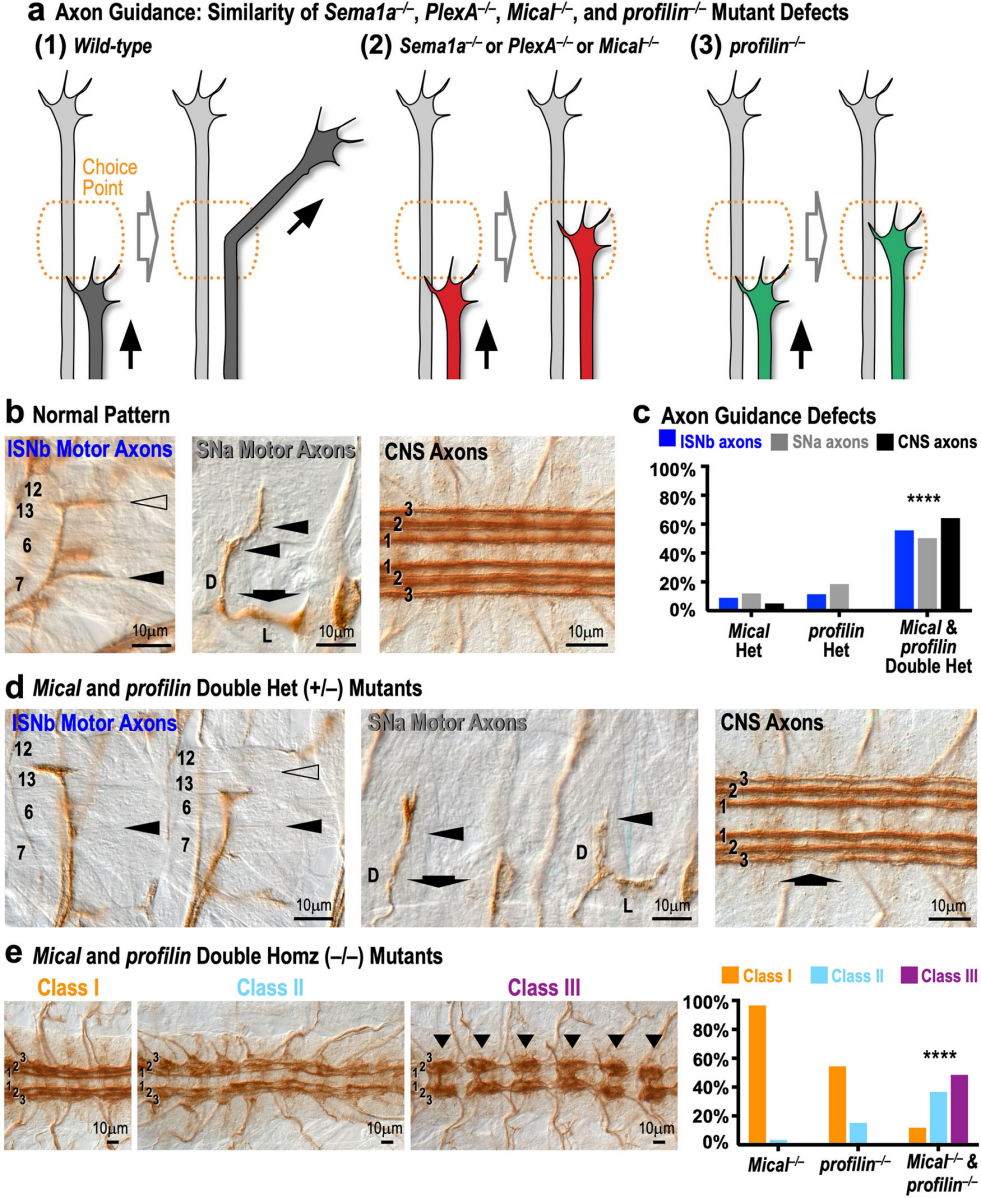
Mical and profilin functionally interact in vivo to guide axons. **a** Similarity of *Sema1a*^*−/−*^*, PlexA*^*−/−*^*, Mical*^*−/−*^, and *profilin*^*−/−*^ mutant axon guidance defects. (1) Axons elongate/fasciculate with other axons (arrow, left). At choice points (orange), axons remodel themselves to grow in new directions (arrow, right). (2) Loss of *Sema1a, PlexA, or Mical* results in axons (red) that stall/don’t respond at choice points and thus do not change their trajectory. (3) Loss of *profilin* also results in axons (green) that stall at choice points and don’t change their trajectory. **b**–**e** *Mical* and *profilin* combine to guide axons in vivo. **b** Normal pattern of motor and CNS axon guidance. ISNb motor axons: defasciculate/separate from their main nerve and project to muscles 6/7 (filled arrowhead) and muscles 12/13 (open arrowhead). SNa motor axons: defasciculate to give rise to a dorsal (D) and lateral (L) branch (arrow)—and dorsal branch axons then make two characteristic turns (filled arrowheads). CNS axons: project within three 1D4-positive longitudinal bundles (1, 2, 3). **c**, **d** Compared to either heterozygote (Het) alone, *Mical*^+/–^ and *profilin*^+/–^ double Hets give rise to significant guidance defects including: absent/abnormal innervation of muscles 6/7 (ISNb, closed arrowhead) and 12/13 (ISNb, open arrowhead), absence of SNa lateral branch (SNa, arrow), failure of SNa axons to turn (SNa, arrowheads), and decreases in axons in the third CNS longitudinal bundle (CNS, arrowhead). *Mical* Het = *Mical*^*I1367*^*/+* (ISNb and SNa, *n* = 100 hemisegments assessed across 10 animals; CNS, *n* = 20 animals). *profilin* Het *=* *chic*^*221*^*/+* (ISNb and SNa, *n* = 70 hemisegments assessed across 7 animals; CNS, *n* = 20 animals)*. Mical & profilin* Double Het *=* *Mical*^*I1367*^*/+ & chic*^*221*^*/+* (ISNb and SNa, *n* = 140 hemisegments assessed across 14 animals; CNS, *n* = 14 animals). ****p < 0.0001; *Χ*^2^ test. **e** *Mical*^*−/−*^ and *profilin*^*−/−*^ double homozygous (Homz) mutants exhibit significantly more severe guidance defects than either homozygous mutant alone—including Class III defects, in which axons are excessively/repetitively bundled together (arrowheads). Scoring system adapted from^41^. Defects: Class I (affecting longitudinal bundle 3), Class II (affecting longitudinal bundles 2 and 3), Class III (affecting longitudinal bundles 1, 2, and 3). *Mical*^−/−^ = *Mical*^*Df(3R)swp2*^*/Mical*^*Df(3R)swp2*^ (*n* = 29 animals; see also^9,40^)*, profilin*^−/−^ *=* *chic*^*221*^*/chic*^*221*^ (*n* = 46 animals; see also^41,51,52^)*, Mical*^−/−^ & *profilin*^−/−^ *=* *Mical*^*Df(3R)swp2*^*/Mical*^*Df(3R)swp2*^ & *chic*^*221*^*/chic*^*221*^ (*n* = 109 animals). ****p < 0.0001; *Χ*^2^ test. **b**–**e** ≥2 independent experiments gave similar results. Source for Fig. 5 are provided as a Source Data file.

In light of our in vitro and in vivo results demonstrating a functional connection between Mical and profilin in regulating actin dynamics and cellular remodeling, it is notable that in general, Mical and profilin exhibit broad overlapping tissue expression patterns and regulate similar types of cellular behaviors in vivo^5,39^. In particular, both profilin and Mical play prominent roles in the development of the nervous system and both of these proteins are localized to axonal growth cones^40–42^. Profilin, similar to Mical, also functionally interacts with the Abl nonreceptor tyrosine kinase to drive neural connectivity^35,41^. We therefore employed the *Drosophila* nervous system as a model to examine if profilin and Mical also work together in vivo to direct the guidance of axons. In particular, axons assemble actin into branched (lamellopodia) and unbranched (filopodia) structures to elongate^43,44^ and they grow in a stereotypic manner, including that they respond at what have been termed “choice points” to remodel themselves—becoming more complex with multiple extending filopodia – and change their direction of growth (Fig. 5a(1)^45–47^). Mical, and its cell-surface binding receptor, Plexin A (PlexA) and PlexA’s ligand, Semaphorin (Sema)-1a, are each required for axons to change their direction at choice points (Fig. 5a(2)^9,40,48,49^)—and previous results support that they use axon–axon repulsion to exert their effects (Supplementary Fig. 6a(1)^9,40,48,49^). Compellingly, previous results support that profilin, working via its role to assist in actin assembly, is also required for axons to emerge from choice points and grow in new directions (Fig. 5a(3), Supplementary Fig. 6a(2)^41,42,50–52^). Therefore, *Sema1a*/*PlexA*/*Mical* and *profilin* mutants give rise to similar types of axon guidance defects but they have not been previously linked together.

To begin to test if Mical and profilin work together in axon guidance, we first used classical dominant genetic interaction assays. In particular, decreasing the levels of *Mical* (*Mical*^+/–^ heterozygous mutant (*Mical* Het)) or *profilin* (*profilin*^+/–^ heterozygous mutant (*profilin* Het)) alone generated no appreciable motor (Intersegmental Nerve b (ISNb) and Segmental Nerve a (SNa)) or central nervous system (CNS) axon guidance defects (Fig. 5b, c). However, we found that when we reduced both the levels of *Mical* and *profilin* at the same time (*Mical* & *profilin* Double Het), significant motor and CNS axon guidance defects occurred (Fig. 5c, d). These defects were similar to those observed in *Mical*^*−/−*^ homozygous mutants and in *profilin*^*−/−*^ homozygous mutants (Fig. 5a(2-3), Supplementary Fig. 7a(1-2)^9,40,41,50,51^). This kind of dosage-sensitive “double heterozygous (transheterozygous)” genetic interaction is very specific and provides strong evidence that two proteins act together in a common pathway in vivo. Furthermore, the transheterozygous interactions between *Mical* and *profilin* that we observed were similar in severity to the transheterozygous interactions that have previously been described between *Mical* and other components of its signaling pathway including *Sema1a*, *PlexA*, *PlexB, Gyc76C, cofilin*, and *Abl*^14,35,40,53–56^. Thus, while previous results (as referenced above) support that Mical and profilin are opposite in the cytoskeletal/guidance effects they promote in vivo, F-actin disassembly/repulsion and F-actin assembly/attraction, respectively, simultaneously reducing them does not result in a canceling out of their effects on growing axons but strongly alters the ability of axons to grow away from choice points.

To further test for a connection between Mical and profilin in the guidance of axons, we next turned to double mutant analysis in CNS axon guidance, which has previously been successfully employed to demonstrate that profilin and other proteins including formins and Abl work together to guide axons (Supplementary Fig. 7a(3)^41,51,52^). Strikingly, our results revealed significant enhancement of the severity of guidance defects in *Mical*^*−/−*^ and *profilin*^*−/−*^ double mutants (Fig. 5e; Supplementary Fig. S6b). Furthermore, the guidance defects in *Mical*^*−/−*^ and *profilin*^*−/−*^ double mutants resembled the double mutant defects that occur between profilin and its other interactors (Supplementary Fig. 7a(3)^41,51,52^). Moreover, the predominant defects in *Mical*^*−/−*^ and *profilin*^*−/−*^ double mutants revealed axons that were severely bundled together (too much fasciculation) within each of the different segments of the embryo, such that the axons were unable to change their trajectory/leave their embryonic segment to project down the cord (Fig. 5e, Class III, arrowheads). These types of guidance defects are consistent with previous results with single mutants alone (as described above) and the hypothesis that in *Mical*^*−/−*^ and *profilin*^*−/−*^ double mutants there is too little F-actin disassembly/repulsion coupled with too little assembly in new directions. Thus, both these transheterozygous and double mutant loss-of-function assays provide strong support that Mical and profilin serve in a connected manner to allow axons to navigate in vivo.

**Fig. 6:**
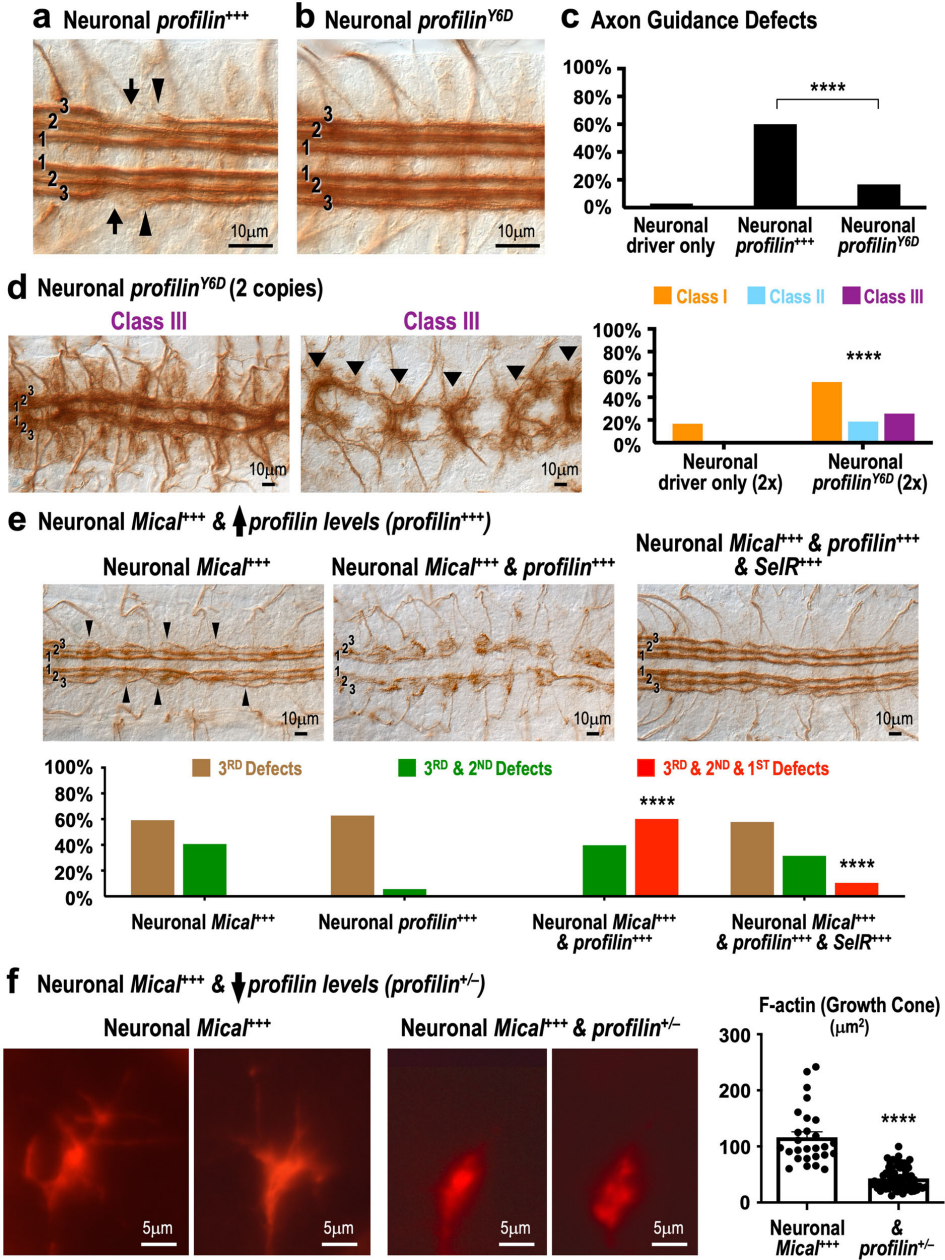
Semaphorin–Plexin–Mical repulsion combines with profilin to drive F-actin remodeling and guidance of growth cones. **a–c** Neuronal overexpression of *profilin* (*ELAV-GAL4/+, UAS:profilin (1M)/+ *(*n* = 100 hemisegments in 10 animals)), but not *profilin*^*Y6D*^ (*ELAV-GAL4/+, UAS:profilin*^*Y6D*^ *(5F)/+ *(*n* = 120 hemisegments in 12 animals)), generates highly penetrant axon guidance defects (arrows), including that axons project away from the CNS (arrowheads). Neuronal driver only = *ELAV-GAL4/+ *(*n* = 100 hemisegments in 10 animals). ****p < 0.0001; *Χ*^2^ test. **d** Neuronal overexpression of high levels (2 copies (2x)) of *profilin*^*Y6D*^ (*ELAV-GAL4*/*ELAV-GAL4, UAS:profilin*^*Y6D*^ *(5F)/UAS:profilin*^*Y6D*^ *(5F)* (*n* = 43 animals)) generates *Mical*^*−/−*^ and *profilin*^*−/−*^ double mutant-like CNS axon guidance defects. Scoring as in Fig. 5e. Neuronal driver only (2x) = *ELAV-GAL4/ELAV-GAL4* (*n* = 12 animals). ****p < 0.0001; *Χ*^2^ test. **e** Neuronal overexpression of *profilin* increases Mical-dependent axon guidance defects and SelR neuronal overexpression rescues these effects. Neuronal *Mical*^*+++*^ (*UAS:Mical/+, ELAV-GAL4/+ *(*n* = 54 animals)): alters the guidance of axons in the 3rd (3) and 2nd (2) CNS longitudinal bundles, including that axons are abnormally separated from one another/present in thinner bundles and project away from the CNS (e.g., arrowheads). See also refs. ^9,56^. These defects contrast to loss of *Mical* (*Mical*^*−/−*^), or *Mical* and *profilin* double mutants (*Mical*^*−/−*^ *& profilin*^*−/−*^), in which axons are more thickly bundled (e.g., Fig. 5e, Class III, arrowheads). Neuronal *Mical*^*+++*^ *& profilin*^*+++*^ (*UAS:Mical/+, UAS:profilin (1M)/+, ELAV-GAL4/+ *(*n* = 88 animals)): significantly increases the severity of guidance defects (****p < 0.0001; *Χ*^2^ test)—now predominantly affecting all three (3, 2, 1) longitudinal bundles (image and graph, red) and generating axons that are even more separated from one another/present in thinner bundles. Neuronal *Mical*^*+++*^ *& profilin*^*+++*^ *& SelR*^*+++*^ (*UAS:Mical/+, UAS:profilin (1M*)/+, *ELAV-GAL4/+, UAS:SelR/+* (*n* = 57 animals)): *SelR* neuronal overexpression significantly suppresses the Neuronal *Mical*^*+++*^ & *profillin*^*+++*^ defects (****p < 0.0001; *Χ*^2^ test), such that a more normal pattern of axons is observed in all three longitudinal bundles (3, 2, 1). Neuronal *profilin*^*+++*^ *=* *UAS:profilin (1M)/+, ELAV-GAL4/+ *(n = 35 animals; see panel **a** and graph). **f** Mical (Neuronal *Mical*^*+++*^=*UAS:Mical*^*ΔPIR*^*/+, RN2-GAL4/+, UAS:*^*GFP*^*Actin*/+ (*n* = 27 growth cones in 5 animals)) significantly increases F-actin (red) remodeling in growth cones^9^—and reducing *profilin* (Neuronal *Mical*^*+++*^ *& profilin*^*+/−*^ = *UAS:Mical*^*ΔPIR*^*/+, RN2-GAL4/+*, *UAS:*^*GFP*^*Actin*/+, *chic*^*221*^*/*+ (*n* = 65 growth cones in 12 animals)) decreases this remodeling. Means ± SEM. ****p < 0.0001; unpaired *t*-test (two-tailed). For **a–f**, ≥2 independent experimen*t*s gave similar results. Source data for Fig. 6 are provided as a Source Data file.

To further examine the connection between Mical and profilin, we next turned to gain-of-function (overexpression) axon guidance assays. In particular, examining the effects of neuronal overexpression of profilin revealed that the guidance defects which resulted from *profilin* overexpression in neurons (Fig. 6a, c) were not only similar to those seen following an increase in the activation of profilin-associated actin elongation-promoting factors, including formins and Ena/VASP^52,57,58^, but were also similar to an increase in Sema–Plex–Mical repulsive signaling (Supplementary Fig. 7a(4)^9,12,49,54,56^). These types of defects were not seen with neuronal overexpression of *profilin*^*Y6D*^ (Fig. 6b, c). Instead, neuronal overexpression of high levels of *profilin*^*Y6D*^ resulted in axon guidance defects that resembled *Mical*^*−/−*^ and *profilin*^*−/−*^ double mutants (Fig. 6d, and compare to Fig. 5e)—as well as double mutant combinations of *profilin* and other components of its signaling pathway (Supplementary Fig. 7a(3)^41,51,52^). These results with the profilin^Y6D^ mutant (Fig. 6d), which has lower affinity for the polyproline residues of actin elongation-promoting factors^37^, were also consistent with the axon guidance defects seen following mutation of actin elongation-promoting factors such as formins and Ena/VASP^52,59–61^. Furthermore, we turned to axon guidance assays that are due to the repulsive effects of Sema–Plex–Mical signaling (Supplementary Fig. 7a (4-5)), and have been used extensively to identify components of Sema–Plex–Mical repulsion^12,14,35,54,56^. Our results revealed that in axons, as in the bristle model system (Fig. 4d, f), raising the levels of *profilin* enhanced the effects of Sema–Plex–Mical-dependent repulsion (Fig. 6e; Supplementary Fig. 7b). These severe defects are similar to what has been described previously for high levels of axon repulsion (Supplementary Fig. 7a (5)^9,54,56^)—such that axons were abnormally separated from one another/present in thinner bundles (i.e., not excessively bundled together as in Fig. 5e, Class III) (Fig. 6e; Supplementary Fig. 7b). These types of defects also resembled those seen following neuronal overexpression of high levels of actin elongation-promoting factors (Supplementary Fig. 7a(5); refs. ^52,57,58^). Likewise, as we saw in vitro with purified proteins (Supplementary Fig. 1) and in the bristle model (Fig. 4g), SelR reversed profilin’s ability to enhance Sema–Plex–Mical-mediated effects (Fig. 6e, Supplementary Fig. 7b). Moreover, similar to what we saw in the in vivo bristle model (Fig. 4c, f), decreasing the levels of *profilin* (*profilin*^*+/–*^) suppressed Mical’s ability to increase F-actin remodeling during growth cone guidance in vivo (Fig. 6f). Therefore, multiple different loss- and gain-of-function interaction experiments indicate that Mical and profilin functionally combine in vivo to direct axon guidance. These results are consistent with our results using purified proteins and the bristle model and further support an interaction between Mical, Mical-oxidized actin, and profilin.

## Discussion

Precise coordination of the assembly and disassembly of the actin cytoskeleton is a critical requirement to shape and reshape cells—including driving their multiple behaviors such as cytokinesis, polarity, motility, guidance, and connectivity. We now find an important convergence point that shifts the balance from a cellular program promoting F-actin assembly to one triggering F-actin disassembly and remodeling. Namely, we find that profilin, an important promoter of formin and Ena/VASP-driven actin assembly, binds F-actin that is post-translationally modified by the F-actin disassembly enzyme Mical. However, unlike unmodified actin, Mical-oxidized actin inhibits profilin-assisted actin assembly. Thus, Mical and profilin interactions with actin result in a synergistic effect: Mical-mediated oxidation impairs the polymerization properties of actin^9–12,14^ and, by binding to profilin, Mox-actin also inhibits formin and Ena/VASP-driven actin assembly. Moreover, compared to unmodified F-actin, Mox-actin filaments exhibit increased instability in the presence of free profilin, further supporting the view that Mical and profilin work in tandem to both destabilize filaments and prevent incorporation of actin monomers into filamentous structures. Our results show that the combined action of Mical and profilin is also critical in vivo to disrupt the status quo actin assembly program, including inducing cytoskeletal remodeling to allow cells and axons to elongate in new directions.

Subcellularly targeted and directed actin assembly and disassembly are required for most if not all cellular events. Actin’s interaction with proteins such as profilin inhibits spontaneous actin nucleation and fuels processive F-actin assembly driven by multiple proteins including formins and Ena/VASP^3,4,19^. Opposed to that, proteins such as the MICAL family of enzymes trigger F-actin disassembly^9–11^, which is governed by specific extracellular cues^35,40^, particular signaling molecules^35,62^, cofilin-mediated F-actin severing^14,15^, and the unusual intrinsic properties of Mox-actin filaments^11^. Our previous results revealed that Mox-actin can disassemble catastrophically^11^, but barbed-end binding factors (such as capping protein) as well as an ATP cap protect Mox-actin filaments from rapid depolymerization^11^. Formins, and Ena/VASP, are also barbed-end binding factors, so we wondered if these positive effectors of actin assembly might block catastrophic disassembly of Mox-F-actin and allow for incorporation of Mox-G-actin into filamentous structures. Our results, however, reveal the opposite: that Mical not only triggers F-actin disassembly and disrupts the ability of actin to polymerize^9–11^, but it also negatively affects the ability of positive effectors of actin assembly to promote polymerization. Thus, our results reveal connections and mechanisms to coordinate the necessary spatiotemporal balance between positive and negative effects on the actin cytoskeleton.

Our results also show that profilin preferentially destabilizes Mox-actin filaments (Fig. 3f). It is well documented that profilin affects actin barbed-end dynamics by increasing the rate of subunits’ dissociation^26^. Modeling studies suggest that binding of profilin to the barbed-end requires reduced flattening of terminal actin subunits to prevent steric clashes. This would induce more of a G-actin-like subunits’ conformation at the barbed-end and increase the rate of monomers’ dissociation. Such conformational change would farther enhance the depolymerization of Mox-actin, which is known to be substantially faster than that of the unoxidized actin even in the absence of profilin^11^. Furthermore, considering the unusual structure of Mox-F-actin^11^, it is also possible that free profilin binds to the barbed ends of Mical-oxidized F-actin with higher affinity compared to that of unoxidized F-actin, therefore, blocking new monomers’ addition^24–26^. We also hypothesize that loss of an ATP cap at the barbed-end of Mox-F-actin may contribute to profilin-induced destabilization of Mox-F-actin (Fig. 3f), which would result in exposure of ADP-bound Mox-F-actin segments that are intrinsically unstable, and can undergo catastrophic collapse^11^. Such intrinsic instability of Mox-F-actin at the barbed ends would be induced if depolymerization of Mox-F-actin is enhanced by profilin and at the same time association of profilin–Mox-ATP-G-actin with barbed ends is greatly inhibited/abolished. Together, our data suggest that the unique intrinsic properties of Mox-actin^10–12^ combined with destabilizing effects of cofilin^14^ and profilin provide a highly effective way of F-actin destabilization.

**Fig. 7:**
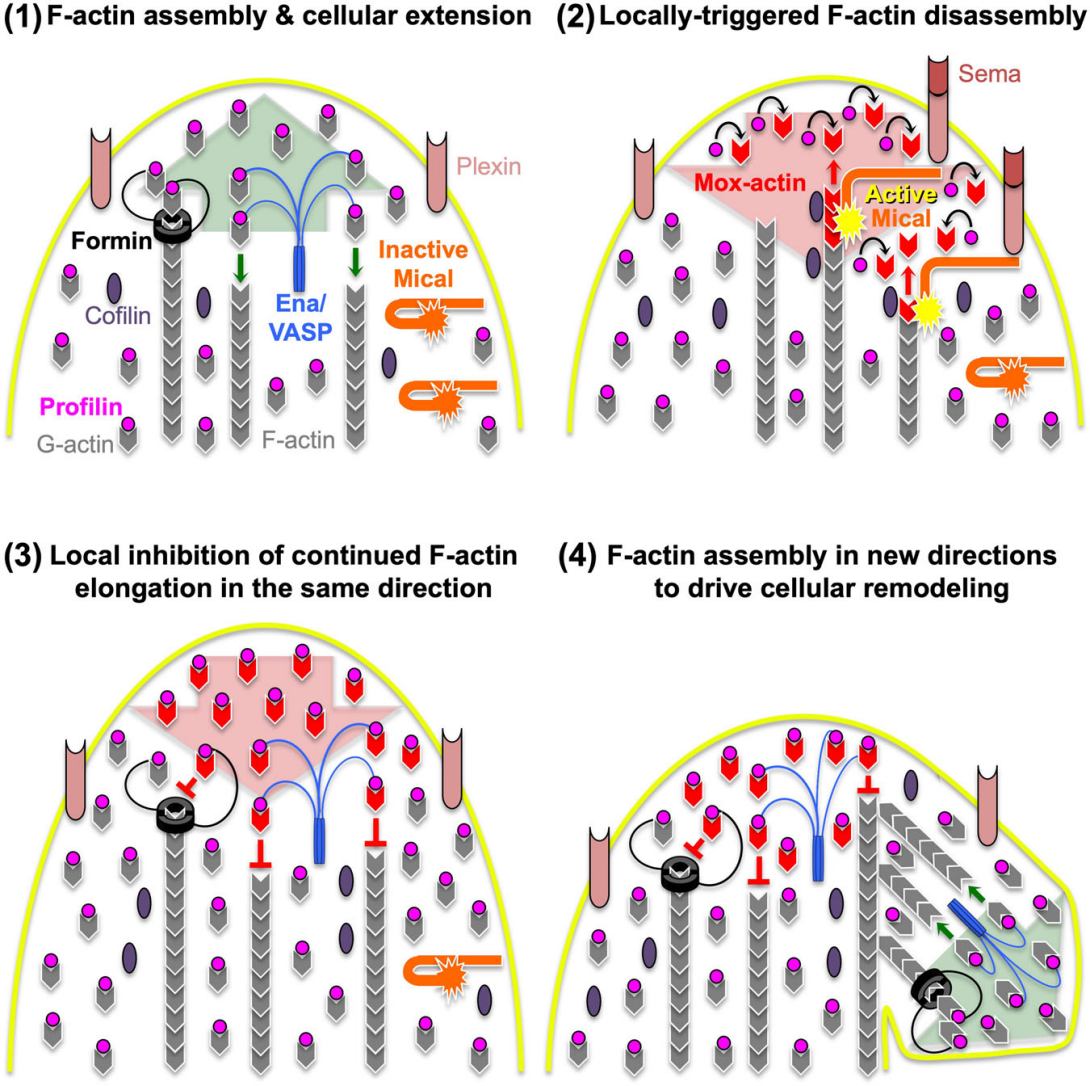
Model of profilin and Mical combining to impair continued F-actin assembly and enhance disassembly and remodeling. Our in vitro and in vivo observations, coupled with previous results (e.g., refs. ^9,10,12,14,32–35,40^), suggest the following working model (depicted in a chronological sequence (1–4)): **(1)** In the absence of Mical activation, profilin binds G-actin and—through its ability to assist in formin and Ena/VASP-driven barbed-end actin polymerization (small green arrows)—acts as a positive effector of actin assembly and cell elongation (large green arrow). **(2)** Mical, in contrast, works in response to negative effectors of cell movement, such as Semaphorin repellents and their Plexin receptors—which locally activate Mical to oxidize and promote F-actin disassembly (small red arrows), which negatively affects cell elongation (large red arrow). This creates a local pool of Mical-oxidized actin (Mox-actin (red)). **(2–3)** At this spatiotemporal point, Mical and profilin’s effects become intertwined to exert a new effect on cytoskeletal and cellular behavior. Namely, Mox-actin (generated in (2)) exerts a secondary effect: by binding to profilin ((2), curved black arrows) and inhibiting profilin’s positive effects on formin and Ena/VASP-driven continued actin elongation ((3), red inhibitory symbols). These Mox-actin–profilin complexes thereby locally inhibit actin elongators in areas where Mical gets activated, stalling continued elongation and cellular growth in the same direction. Additionally, profilin, through its ability to interact with Mox-F-actin, enhances Mox-F-actin disassembly. Thus, these combined effects of Mical and profilin give rise to local subpopulations of disassembled F-actin and paused/slow growing barbed ends ((3), large red arrow). **(4)** Since profilin is ubiquitously localized, it also binds to unmodified actin, which is located outside of regions where Mical is actively disrupting actin filaments/elongation. Profilin, then, supplies this unmodified actin to relieve Mox-actin-induced inhibition of actin elongation-promoting factors, assisting in new branch formation/cellular remodeling by inducing actin polymerization (e.g., small green arrows) and elongation in new directions (large green arrow). For simplicity/to aid in visualization, formins and Ena/VASP’s association with the cell membrane (yellow) is not illustrated. Similarly, some molecular components are not illustrated in each panel of (1–4). Diagram modified from ref. ^36^.

Our results also paint a fuller picture of the mechanisms allowing for a balance between positive and negative effects on cellular behaviors such as outgrowth/motility and navigation. In particular, in the high-resolution bristle cell model^30,31^, *profilin* “knockout” mutants generate defects that include short bristles with filaments/bundled filaments that are abnormally positioned and thinner than normal^32–34^; supporting the idea that profilin is critical for actin assembly in vivo. *Mical* “knockout” mutants, in contrast, result in too much F-actin accumulation in bristles, showing that Mical is critical for actin disassembly in vivo^9,12^. Therefore, profilin and Mical’s effects on actin dynamics in vivo are generally opposite in function. However, when Mical and profilin are combined together, a new effect ensues that not only disrupts continued actin assembly and cell elongation, but allows cells to remodel themselves and extend branches in new locations and directions. Therefore, based on our results we propose a working model that Mical–profilin cross-talk events lead to cessation of ongoing F-actin assembly, enhanced F-actin disassembly, and cellular remodeling in new directions (Fig. 7). Specifically, activation of Mical results in local generation of Mox-actin (Fig. 7, (1)-(2)). Profilin then: (i) binds to these Mox-actin monomers, which blocks their reincorporation into filaments, and (ii) acts as a disassembly-assisting factor to enhance destabilization of Mical-oxidized filaments (Fig. 7, (2)-(3)). Subsequently, profilin assists actin elongation-promoting factors (formins and Ena/VASP) in using unmodified G-actin to build new F-actin structures, thereby promoting local remodeling/outgrowth in new directions (Fig. 7, (4)). Interestingly, although the molecular and biochemical mechanisms have not been delineated, such cellular remodeling and plasticity events have been noted as the response of cells to repellents such as Semaphorins, Ephrins, and Slits (reviewed in ref. ^45^): such that F-actin disassembly and inhibition of actin assembly occurs at the tips of cells, but new branches then form to send cells and axons in new, more permissive, directions (e.g., refs. ^9,63–67^). Yet, since cellular repulsion has long been associated with F-actin disassembly (reviewed in ref. ^45^), the previously observed involvement of positive effectors of F-actin assembly as a part of cellular repulsion (e.g., refs. ^61,68–72^) has generally been viewed as enigmatic and confusing. Our results now uncover a means by which negative and positive effectors of F-actin work “hand-in-hand” in these events, thereby providing important insights into the molecular and biochemical mechanisms underlying cellular repulsion.

In conclusion, motile cells and growing axons utilize poorly understood mechanisms to finely tune their advancement so that they are able navigate complex routes and reach their destinations. Previous work supports that positive and negative growth/extension/guidance signals underlie these directional changes by enigmatically pausing ongoing advancement and inducing growth/elongation in new directions. Our results now support a mechanism that allows for the integration and balance of such positive and negative signals at the level of the cytoskeleton to stall ongoing advancement and allow growth/extension in new directions. Given the broad expression patterns and physiological and pathological roles for profilins, formins, Ena/VASPs, and MICALs, the interactions we have observed herein are likely to be widespread and play important roles in physiological and disease processes.

## Methods

### Protein preparations

Rabbit skeletal actin (RSA) (see ref. ^11^ and references therein) and profilin^73^ were prepared using established protocols. Actin was labeled with pyrene maleimide and Alexa488SE according to established protocols^11^. In particular, RSA was polymerized by addition of 100 mM KCl, pelleted, and shifted into the labeling buffer (50 mM PIPES, pH 6.8, 50 mM KCl, 0.2 mM CaCl_2_, 0.2 mM ATP) on dialysis. The resulting F-actin was labeled overnight at 4 °C with three-fold molar excess of Alexa488SE dye followed by pelleting, depolymerization, and gel filtration. Actin oxidized by Mical enzymes was prepared by incubating F-actin with Mical^RedoxCH^ at 50:1 molar ratio in the presence of 0.2 mM NADPH followed by the removal of the residual Mical enzyme by gel filtration (Superdex S200 16/60 column, Amersham Biosciences)^11^. Alternatively, gel-filtered actin was dialyzed against G-buffer (GB2: 5 mM Tris, 0.2 mM CaCl_2_, 0.2 mM ATP, 0.5 mM DTT pH 8) and polymerized for 1 h at room temperature (RT) by adding 2 mM MgCl_2_ and 50 mM KCl. F-actin (10 µM) was oxidized in the presence of 0.1 µM Mical and of 0.3 mM NADPH for 10 min at RT. Mox-actin was dialyzed overnight against GB2 supplemented with 2 mM β-mercaptoethanol instead of DTT. Any residual F-actin or/and actin aggregates were pelleted in TLA 110 rotor (90,000 rpm (average of 338,000 × g), 20 min, 4 °C). The resulting supernatant was mixed with 0.25 ml Ni-NTA resin equilibrated with GB2 and 2 mM β-mercaptoethanol to remove the residual Mical enzyme. After 40 min incubation at 4 °C, the flow through was collected and dialyzed overnight against GB2. Judging from the critical concentration measurements and subtilisin digestion, both protocols yield the same quality Mox-actin preparations. Labeled Mox-actin was prepared under polymerizing conditions (5 mM Tris, 0.2 mM CaCl_2_, 0.5 mM DTT, 0.2 mM ATP, 2 mM MgCl_2_, 50 mM KCl, pH 8) by incubating it with Mical at 70:1 (actin:Mical) molar ratio in the presence of 100 μM NADPH for 1 h at RT. The resulting actin was dialyzed overnight against GB2 then centrifuged (TLA100 rotor, 90,000 rpm (average of 312,530 × g), 30 min, 4 °C)^14^.

The plasmid containing human INF2-FFC gene fragment was a kind gift from Dr. Henry Higgs (Dartmouth College). INF2 was expressed as a N-terminal GST-fusion protein in Rosetta2(DE3) cells at 16 °C for 18 h. Expression was induced with 0.5 mM IPTG. INF2 was purified using a combination of affinity chromatography and gel filtration. Specifically, cells were lysed in the extraction buffer (50 mM Tris, 500 mM NaCl, 5 mM EDTA, 1 mM DTT, 0.2 mM PMSF) supplemented with leupeptin, pepstatin, and trypsin inhibitor and spun down for 1 h (113,600 × g) at 4 °C. The resulting supernatant was supplemented with 0.1% thesit. GST-tagged INF2 construct was bound to glutathione affinity column (Glutathione-Sepharose 4B (GE Healthcare)) and washed with the following buffers: (1) WB1: 10 mM Tris (pH 8) 250 mM NaCl, 1 mM EDTA, 1 mM DTT, 0.05% thesit; (2) ATP wash: 20 mM Hepes (pH 7.4), 20 mM MgCl_2_, 5 mM ATP, 150 mM KCl; (3) WB2: 10 mM Tris (pH 8), 250 mM NaCl, 1 mM MgCl_2_, 1 mM EGTA, 1 mM DTT, 0.05% thesit; and (4) WB2 containing universal nuclease followed by additional 50 ml of WB2. On-column cleavage of the GST tag was carried out either in WB1 or in the cleavage buffer (50 mM Tris (pH 8), 250 mM NaCl, 0.2 mM EDTA, 0.5 mM DTT). Cleaved formin construct was further purified on gel-filtration column (HiLoad 16/60 Superdex 75 (Amersham Biosciences)) equilibrated with 20 mM Hepes (pH 7.5), 200 mM NaCl, 1 mM DTT, 0.2 mM PMSF, 0.01% NaN_3_. Purified protein was concentrated and dialyzed against 1×KMEH7 (10 mM HEPES (pH 7), 1 mM EGTA, 1 mM MgCl_2_, 50 mM KCl) supplemented with 1 mM DTT. After the final centrifugation step (TLA 110, 80,000 rpm (average of 267,000 × g), 20 min, 4 °C), the protein was aliquoted and stored at −80 °C. mDia2-FFC and mDia2-FF constructs were expressed in Rosetta2(DE3) cells and purified using glutathione affinity column (Glutathione-Sepharose 4B (GE Healthcare))^74,75^. Additionally, two rounds of gel-filtration chromatography on HiLoad 16/60 Superdex 75 column were performed for the GST-tagged mDia2-FF construct to separate lower molecular weight contaminants. The protein was recovered in 2×KMEH7 buffer supplemented with 2 mM DTT and concentrated. After the final centrifugation step (TLA 110, 80,000 rpm (average of 267,000 × g), 20 min, 4 °C), the GST-mDia2-FF was supplemented with glycerol (50% final) and stored at −20 °C. *Drosophila* formin cappuccino (Capu) was a generous gift from Dr. Margot Quinlan (UCLA). Ena/VASP-SNAP was a generous gift from David Kovar (University of Chicago).

*Drosophila* melanogaster SelR gene (codon-optimized for expression in *E. coli*) was cloned into the modified pGEX-6P2 vector. The resulting protein construct had an N-terminal GST tag followed by the PreScission protease cleavage site and C-terminal 6-His tag downstream of the thrombin cleavage site. SelR was expressed in Rosetta2 cells cultured in LB broth supplemented with 100 µg/ml ampicillin, 33 mg/ml chloramphenicol, and 20 µM ZnCl_2_. SelR expression was induced with 1 mM of IPTG and carried out at 18 °C overnight. The cells were harvested and frozen at −80 °C. To purify SelR, cells were resuspended in buffer D (21.4 mM Na_2_HPO_4_, 3.8 mM KH_2_PO_4_, 300 mM NaCl, 0.2 mM PMSF, and 5 mM β-mercaptoethanol, pH 7.5). The cells were sonicated and centrifuged for 30 min at 4 °C at 20,428 × g. The supernatants were mixed with the glutathione-Sepharose 4B (GE Healthcare) beads and incubated for 30 min on a nutator at 4 °C. Before the elution step, SelR-bound beads were subjected to a series of washes including buffer D supplemented with 10% glycerol, buffer D supplemented with 1 M NaCl, and 10 mL wash containing 1 μL of Universal Nuclease in buffer D (ThermoFisher, PI88701). Protein was eluted with Tris buffer containing 10 mM glutathione (pH 8) and GST tag was cleaved with PreScission protease on dialysis overnight. Cleaved SelR was loaded onto a 1 mL HisTrap Ni2 + column (GE Healthcare) and eluted with a 0–100% gradient (25CV) of imidazole in a following buffer: 10 mM Tris, pH 8, 300 mM NaCl, 5% glycerol, 3 mM β-mercaptoethanol, 0-250 mM imidazole. The protein was concentrated and dialyzed overnight against the following buffer: 10 mM Hepes, 1 mM MgCl_2_, 50 mM KCl, 0.2 mM EGTA, 0.5 mM TCEP, and 1 mM DTT. Before freezing, the preparation was precleared by a high-speed spin (302,142 g, 20 min at 4 °C).

### Assessing actin–profilin binding via changes in intrinsic fluorescence

Profilin binding was monitored by a change of intrinsic tryptophan fluorescence of actin^16^. Specifically, changes in intrinsic fluorescence were monitored using PTI spectrofluorimeter, with excitation wavelength set at 295 nm and emission at 330 nm. Actin preparations were dialyzed against (and later diluted with) 5 mM PIPES buffer (pH 7, 0.2 mM CaCl_2_, 0.2 mM ATP, 0.5 mM DTT). Concentrated profilin stock was titrated into 0.15 μM of actin (V = 2.5 ml). In our experiments, actin dilution with profilin did not exceed 1–2% and, therefore, actin concentration was considered constant throughout the titration. For each experiment we first titrated profilin into the buffer (linear dependences were obtained). Then the same profilin stock was titrated into actin containing samples. Calculations were performed as follows (see ref. ^16^): ΔF = F_P_ − (F_AP_ − F_A_) where ΔF is the difference in intrinsic fluorescence between profilin alone (F_P_) and profilin–actin complex (F_AP_) and F_A_ corresponds to the fluorescence of actin alone. The resulting binding curves were normalized using the fluorescence values at the highest profilin concentrations. Presentation of these and other biochemical results was done using SigmaPlot (Ver#11).

### High-speed pelleting assays

Unless stated otherwise, high-speed centrifugation was carried out in TLA100 rotor at 90,000 rpm (average of 312,530 × g), 4 °C for 20 min. Resulting supernatants and pellets were analyzed using SDS-PAGE and staining with Coomassie Blue. Fiji (ImageJ; Ver #1.5.3a) software was used for gel quantification. Source data/uncropped gels are provided as a Source Data file.

### Pyrene fluorescence assays

Unoxidized Mg-ATP-G-actin (2.5% pyrene-maleimide labeled) was used as a reporter in pyrene assembly assays. It should be noted, that in our preliminary tests using Mox- vs unoxidized pyrene-labeled G-actin as a reporter showed the same trends.

Ca-ATP-G-actin was converted into Mg-ATP-G-actin by incubation with ME exchange buffer (0.05 mM MgCl_2_ and 0.2 mM EGTA) for 3 min. Polymerization reactions were started by mixing of 25 μl of 5 × concentrated actin/actin–profilin with 100 μl of 1.25 × concentrated mixtures of all other reagents. The final concentrations are indicated in the figure legends. Note, that ionic strength of the polymerization buffer was slightly lowered (0.8×KMEH7) in the pyrene assembly assays to slow down spontaneous actin nucleation and allow for more accurate assessment of the contribution of the formin-mediated nucleation. Changes in pyrene fluorescence signal over time (Pyrene fluorescence (arbitrary units (A.U.)) were monitored using a TECAN microplate reader and for clarity of presentation, the fluorescence intensity values are shown in the graphs as reduced by a factor of 1000 (see Source data file for Raw data). The graphs in Figs. 1d, 2a, e, f, 3a, c, d show averages of 2–3 technical replicates (i.e., samples run within the same plate, at the same time) that were obtained in one representative independent experiment. As noted in the figure legends, each experiment was also repeated 2–3 independent times (i.e., *n* = 2–3 separate experiments with similar results). In some cases, actin samples were removed from the microplate after monitoring the changes in pyrene fluorescence and subjected to high-speed pelleting (see above).

Seeded bulk actin polymerization assays were carried out as follows. F-actin seeds were prepared by incubating unoxidized F-actin with phalloidin at 50:1 actin:phalloidin ratio. Such ratio was chosen to prevent depolymerization of the seeds over time and at the same time to ensure that there is no free phalloidin in reaction mixtures because it can stabilize Mox-actin. Polymerization buffer and the seeds were mixed and incubated 15 min at RT to allow for filament ends to anneal. If both seeds and formin were used, formin was added into the mixtures 15 min after the seeds followed by additional 15 min incubation on the nutator at RT.

### Limited proteolysis with subtilisin

Limited proteolysis with subtilisin was carried out with monomeric actin^14^ with the following modification: the w/w ratio of 1:750 (subtilisin-to-actin) was used to digest actin samples.

### TIRF microscopy

Time lapse TIRF microscopy was performed according to the established protocol^11^. In particular, untethered actin filaments were imaged in ~12 μl flow chambers. First, two (2) chamber volumes (CV) of 1% Pluronic F127 solution (Sigma, P2443) were introduced and incubated for 3 min. Next, flow chambers were equilibrated with 2CV of 1×TIRF imaging buffer (10 mM HEPES, 1 mM MgCl_2_, 50 mM KCl, 0.2 mM EGTA (pH 7) supplemented with 50 mM DTT, 0.2 mM ATP, 20 mM glucose, 0.5% methyl cellulose). For seeded elongation assays, the above equilibration buffer was supplemented with 4.8 nM of rhodamine phalloidin-stabilized F-actin seeds (50:1, actin:phalloidin). To prepare the samples, actin–profilin complexes were incubated with ME exchange buffer for 3 min at RT and mixed with the other reaction components. Reaction mixtures (50 μl) were then introduced into the flow chambers. Final reaction mixtures were in 1 × TIRF imaging buffer supplemented with 0.05 mg/ml casein, 0.25 mg/ml glucose oxidase, and 50 μM catalase to minimize photobleaching during the imaging. Actin concentrations were 0.5 µM and 1.4 µM for unoxidized and Mox-actin, respectively (0.4 µM above their critical concentrations). Profilin-to-actin ratios were 3:1 for both actin forms. In the samples containing mDia2-FFC its final concentration was 1.5 nM.

To assess nucleation efficiency of unoxidized and Mox-actin in the presence of mDia2 formin, the reaction mixtures (3 μM of actin (15% Alexa488SE) with and without 30 nM mDia2-FFC) were incubated for 9 min at RT and then diluted 150 fold in 1 × KMEH7 buffer supplemented with 1 μM of phalloidin. Diluted mixtures were applied onto a polylysine-coated surface^74^ and random fields were imaged using DMI6000 TIRF microscope (Leica).

### Pull-down experiments with GST-mDia2-FF construct

GST-tagged mDia2-FF or GST were incubated for 1 h at RT with untagged proteins of interest (5 µM Mox- or unoxidized actin and 15 µM profilin) in LT-KMEH7 buffer (10 mM HEPES, pH 7, 1 mM EGTA, 1 mM MgCl_2_, 50 mM KCl, 1 mM DTT, 0.2 mM ATP, 0.5 mM Latrunculin A, 0.5 mM thesit, and 0.75 mM Tris, pH 8). These mixtures also contained 3% of glycerol (contributed by formin’s stocks) and 2% DMSO (contributed by latrunculin stocks). No actin polymerization under our experimental conditions was confirmed in high-speed pelleting experiments (TLA100 rotor, 80,000 rpm (average of 247,000 × g), 4 °C, 20 min). Protein mixtures were applied onto glutathione-Sepharose beads equilibrated with LT-KMEH7 (10 mM HEPES, pH 7, 1 mM EGTA, 1 mM MgCl_2_, 50 mM KCl, 1 mM DTT, 0.2 mM ATP, 0.5 mM Latrunculin A, and 0.5 mM thesit) and nutated for 2–2.5 h at 4 °C while protected from light. Beads were pelleted by centrifugation (2000 × g, 1 min, 25 °C) and then washed one time with five bead volumes of LT-KMEH7 (95 µl). Proteins were eluted with warm (60 °C) sample loading buffer (1/5th of input volumes). All inputs and eluates were analyzed by SDS-PAGE. Gels were stained with Coomassie Blue. Quantification of the protein bands was done using ImageJ software (NIH; Ver #1.5.3a).

### Genetics, molecular biology, and transgenic lines

Mical pUAST flies, SelR pUAST flies, SelR^C124S^ pUAST flies, and ^HA^PlexA pUAST flies were generated in previously published work^9,12,40^. *UAS:profilin* (*chickadee*) lines were obtained from Lynn Cooley (Yale University), including lines *UAS-chic.C* and *UAS-chic.J*^33^ (these have also been called *UAS-chic78.3* and *UAS-chic36.5*) and both showed similar effects on Bristle *Mical*^*+++*^. We also generated new wild-type *Drosophila UAS:profilin* (*chickadee*) lines as a match to the *Drosophila UAS:profilin* (*chickadee*) Y6D mutant. To do this, plasmids containing wild-type *Drosophila profilin* (*chickadee*) and its Y6D mutant were a kind gift from Dr. Margot Quinlan (UCLA). Then, to generate *Drosophila profilin* (*chickadee*) pUAST flies, the vector containing *Drosophila profilin* (*chickadee*) was subjected to PCR with the following primers 5’-agctgaattcatgagctggcaagattatgt-3’ and 5’-agcttctagactagtacccgcaagtaatca-3’, which contained an EcoRI restriction enzyme site at the 5’ end and an XbaI site at the 3’ end (Supplementary Table 1). Following digestion with EcoRI and XbaI, the resultant *Drosophila profilin* cDNA was moved into the pUAST vector to generate the transgenic flies. To generate *Drosophila profilin*^*Y6D*^ pUAST flies, a vector containing *Drosophila profilin*^*Y6D*^ (*chickadee*^*Y6D*^) was subjected to PCR with the following primers 5’-agctgaattcatgagctggcaagattatgt-3’ and 5’-agcttctagactagtacccgcaagtaatca-3’, which contained an EcoRI restriction enzyme site at the 5’ end and an XbaI site at the 3’ end (Supplementary Table 1). Following digestion with EcoRI and XbaI, the resultant *Drosophila profilin*^*Y6D*^ cDNA was moved into the pUAST vector to generate the transgenic flies. *Drosophila* embryo injections were done by BestGene, Inc. All transgenic fly lines of the same genotypes showed similar bristle defects when expressed with *Mical* and the bristle-specific *B11-GAL4* driver. Effects on bristle morphology on their own (without Mical) were also seen in *UAS:profilin* (*chickadee*) lines including *UAS-chic.C* and *UAS-chic.J* (Supplementary Fig. 4g), although the penetrance of these effects differed somewhat among these lines (i.e., in some of these *UAS:profilin* lines, one copy, while enhancing Mical, did not show defects on their own, and required multiple copies of expression in bristles to see morphological effects on their own; other lines showed morphological defects when one copy was expressed in bristles). Note that for the experiments in Fig. 4, the *UAS:profilin* and *UAS:profilin*^*Y6D*^ lines that we utilized did not show defects on their own (i.e., we chose these weaker expressing lines, with no effects on their own, so we could directly look at effects on Mical). Likewise, the loss-of-function/”knockout mutant *chic* alleles we used exhibit no bristle defects in the heterozygous (+/–) condition (*chic* mutant*/+*). All other mutant lines were obtained from the Bloomington Stock Center except *Mical* point mutation stocks (kind gifts from Hermann Aberle) and B11-GAL4 (a kind gift from John Merriam).

### In vivo analysis of bristle cell morphology and F-actin organization

In vivo analysis of morphology and F-actin organization was done using previously developed approaches and *Drosophila* bristle cells^9,12,14,35,36^. In particular, because we have used Mical’s effect on bristle cells to screen for genetic modifiers of that effect, we have been using a single genetic line and similar types of bristle cells to make it easier to look for genetic modifiers (i.e., the effect that we see in Fig. 4b of this paper is similar to the effect we see in Fig. 1F of ref. ^35^)—and a similar nomenclature is used herein such that one copy of the bristle-specific GAL4 driver, *B11-GAL4*, and one copy of the Mical transgene, *UAS:Mical*, (we call this genotype Bristle *Mical*^*+++*^ (e.g., see ref. ^35^)) are used in Fig. 4. In Supplementary Fig. 5a-c, two copies of the *UAS:Mical* transgene and one copy of the *B11-GAL4* driver are used. For analysis of bristle morphology and F-actin organization in *Drosophila* pupa, pupae were collected and genotyped with the aid of Tb balancers^9,12,14,35,36^. Pupae were then placed on double-sided tape on a slide kept inside a moist petri dish. Pupae were kept in a 25 °C incubator until they reached the desired stage for imaging. Pupae were then removed from their outer cases with Dumont #5 forceps and mounted, keeping the dorsal side up, in depression well slides and immersed in VectaShield Mounting Medium (Vector Laboratories). Bristles were then imaged with the aid of Zeiss LSM710 confocal microscope, and images were stacked using Zen lite software (Zeiss; Ver #8.1). For analysis of bristle morphology in adults, recently emerging offspring were collected and examined under a dissecting microscope (Leica Stereo Zoom S8 APO). Bristles were examined for morphological alterations including branching, bending, and alterations to their tips, and were imaged and drawn with the aid of a Zeiss Discovery M^2^ Bio stereomicroscope, a Zeiss Axiocam HR camera, motorized focus and zoom, and three-dimensional reconstruction software (Zeiss Axiovision software (Ver #4.8.2), Extended Focus software (Ver #1; a kind gift from Bernard Lee), and Microsoft Powerpoint software (Ver #16.49)). The number of branches per bristle was counted on posterior scutellar bristles and represented as the mean number of branches per bristle (±the standard error of the mean (SEM)). The length of the bristle branch was taken from the distal-most branch and was measured by drawing a line on the bristle image and measuring the length of the line using Image J software (NIH; Ver #1.5.3a). Measurements were taken from distinct samples (different bristle cells in different animals) as noted in the figure legend. For genetic interaction analysis, flies of the appropriate genotype were mated to one another, kept in a 25 °C incubator, and then adult offspring were examined^9,12,14,35,36^ for alterations to their posterior scutellar bristles, thereby allowing precise comparison between single cells from animal to animal. Similar results were seen with multiple different “knockout” mutants and overexpression alleles. It should also be mentioned, since profilin and its effects on F-actin are required to allow bristles (e.g., ref. ^32^) and axons (e.g., ref. ^41^) to extend normally, our experimental design was to decrease (but not eliminate) the levels of profilin, so that we could test profilin’s requirement for Mical’s effects without having the overriding strong defects in F-actin polymerization/morphology and lethality that are the result of completely removing endogenous profilin. Thus, our experiments in Figs. 4c, f, and 6f and Supplementary Fig. 5b-c still contain some endogenous profilin (i.e., it is a heterozygous *profilin* background, *profilin*^*+/–*^; ~50% reduction in profilin levels). We therefore followed a similar experimental design for our studies with profilin^Y6D^ (i.e., wild-type profilin is still present in the background in which we express profilin^Y6D^). We also used a similar strategy for expressing profilin^Y6D^ in our analyses of axon guidance.

### In vivo analyses of axon guidance and F-actin organization in growth cones

In vivo analysis of axon guidance was done as previously developed using motor and CNS axons in the *Drosophila* embryonic nervous system (e.g., refs. ^14,35,40,56^). In particular, flies of the appropriate genotype were mated to one another, kept in a 25 °C incubator, and then embryos were collected, processed, fixed, genotyped, and staged. Embryos were then incubated with an N-CAM/Fasciclin II antibody (1:4; Cat# 1D4 anti-fasciclin II supernatant, Developmental Studies Hybridoma Bank; ref. ^50^), an HRP-conjugated secondary antibody (1:500; 115-035-003, Jackson), and then following staining, embryos were incubated and dissected in 70% glycerol. Double heterozygotes and neuronal overexpression of one copy of profilin or profilin^Y6D^ were examined in a detailed manner and quantified for alterations to axon guidance using previously developed criteria (e.g., refs. ^9,14,35,40,48,49,56^ and see Results) and a Zeiss Axioimager microscope equipped with DIC optics, a Zeiss Axiocam HR camera, and Zeiss Axiovision software (Ver #4.8.2). Likewise, Semaphorin–Plexin–Mical repulsive CNS axon guidance defects were quantified for alterations as previously defined (e.g., refs. ^12,14,35,56^ and see Results), where embryos were examined for defects in CNS axonal pathfinding including discontinuous or missing first, second, or third CNS longitudinal connectives and/or axons crossing the midline or projecting inappropriately into the periphery. Double mutant combinations and related single mutant analyses (Fig. 5e and Supplementary Fig. 6b) and neuronal overexpression of two copies of profilin^Y6D^ were examined for defects in axon guidance and quantified using criteria similar to what others have previously employed^41,51,52^. In particular, the Class scoring system for these defects was based on^41,52^ such that: Class I defects were scored if the third longitudinal was affected (i.e., disorganized, disrupted, missing, and/or incorrectly projecting across the midline or into the periphery) but the 1st and 2nd longitudinals had no defects/were intact, Class II defects were scored if the 2nd longitudinal was now also affected (i.e., disorganized, disrupted, missing, and/or incorrectly projecting across the midline or into the periphery) but the 1st longitudinal was intact, and Class III defects were scored if all three longitudinals were affected (i.e., disorganized, disrupted, missing, and/or incorrectly projecting across the midline or into the periphery). All embryos collected of the correct genotype were scored as either normal (not demarcated on the graph in Fig. 5e and Supplementary Fig. 6b) or one of the three different classes of defects and those percentages are reflected in the graphs. Our observations with *profilin*^*−/−*^ (*chic*^*221*^/*chic*^*221*^) in which we observed disorganized longitudinal connectives with occasional gaps is similar to that previously described for this allele^51,52^, and is similar to defects reported for other combinations of *chic* alleles^41,51^. Different combinations of *Mical* alleles also exhibit the same types of disorganized longitudinal connectives^9^ and we observed similar defects when *profilin* was combined with the different *Mical* alleles we tested including *Mica*^*I1367*^ (from ref. ^76^), *Df(3* *R)swp2* (from ref. ^40^), and *Mical*^*K1496*^ (from ref. ^76^). Note that *Mical*^*Df(3R)swp2*^ is a small deficiency that removes *Mical* and several adjacent genes and shows similar axon guidance and synaptogenic defects as mutations in *Mical* alone (e.g., refs. ^9,12,40,76^). Analyses of actin reorganization within growth cones in vivo was done by employing the developing embryonic *Drosophila* nervous system and aCC/RP2 pioneer motor neurons utilizing the RN2-GAL4 driver (e.g., refs. ^9,77^). In particular, the area of the actin containing growth cones in different genetic backgrounds was done in age-matched embryos by normalizing to a similar level of fluorescence intensity and measuring the area of GFP-actin immunostaining (Rabbit anti-GFP, 1:1000, A-11122, ThermoFisher; HRP-conjugated anti-rabbit IgG secondary antibody, 1:100, G-21234, ThermoFisher; Alexa Fluor 594 tyramide, B40957, ThermoFisher) in the tips of axons using Zeiss Axiovision software (Ver #4.8.2) following capturing of the images with a Zeiss Axioimager upright fluorescence microscope and a Zeiss Axiocam HR camera. Measurements were taken from distinct samples (different growth cones in different animals) as noted in the figure legend.

### Statistics and reproducibility

For each representative protein purification, image, gel, immunoblot, graph, or in vivo experiment, the experiments were repeated at least two separate independent times and there were no limitations in repeatability. No statistical method was used to predetermine the sample size, which was based on what is published in the field. Differences between experimental and control animal conditions were highly notable, with relatively little variability and so the sample size was larger than needed to ensure adequate power to detect an effect. Animal studies were based on pre-established criteria to compare against age-matched animals. Animal experiments were not randomized. Animals of the correct genotype were determined and those collected of that genotype were included as data. Likewise, for biochemical experiments, samples were grouped together based on experimental conditions and collected data points for those experiments are presented. For all genetic experiments, the genotype needed to be determined based on different *Drosophila* genetic/chromosome markers, so blinding was not employed. Likewise, for the biochemical experiments, different proteins and reagents for the particular data set needed to be added and then analyzed using specific technical approaches and expertise, and so blinding was not employed. For both genetic and biochemical experiments, differences between the control and experimental conditions were highly notable and reproducible in both biological and technical replicates. For each graph, the statistical test used, the value of n, what n represents, and the p value for each comparison are stated in the figure legend. Graphs show mean ± SEM or the percentage of animals with that defect. Statistical methods used were based on what is standard in the field and no statistical tests were used to determine whether the data met assumptions of the statistical approach. All statistical analyses were performed using GraphPad Prism (Ver #8.4.3). A *p* value of *p* > 0.05 is not considered statistically significant. Single asterisk (*) indicates *p* < 0.05, double asterisks (**) indicate *p* < 0.01, triple asterisks (***) indicate *p* < 0.001, quadruple asterisks (****) indicate *p* < 0.0001. To the best of our knowledge the statistical tests are justified as appropriate.

### Reporting summary

Further information on research design is available in the Nature Research Reporting Summary linked to this article.

## Supplementary information

Supplementary Information

Reporting Summary

## Data Availability

All relevant data supporting the key findings of this study are available within the article and its Supplementary Information files or from the corresponding author upon reasonable request. Source data are provided with this paper.

## Source data

Source Data
