# Supplementary material for: Profilin and Mical combine to impair F-actin assembly and promote disassembly and remodeling
Source: Nat Commun. 2021 Sep 20;12:5542. doi: 10.1038/s41467-021-25781-3 (PMC8452626; doi:10.1038/s41467-021-25781-3)
Supplement: Supplementary file 1 — Supplementary Information [file 41467_2021_25781_MOESM1_ESM.pdf]

Supplementary Material for

Profilin and Mical combine to impair F-actin assembly and promote disassembly and remodeling

Elena E. Grintsevich, Giasuddin Ahmed, Anush A. Ginosyan, Heng Wu, Shannon K. Rich, Emil Reisler, and Jonathan R. Terman

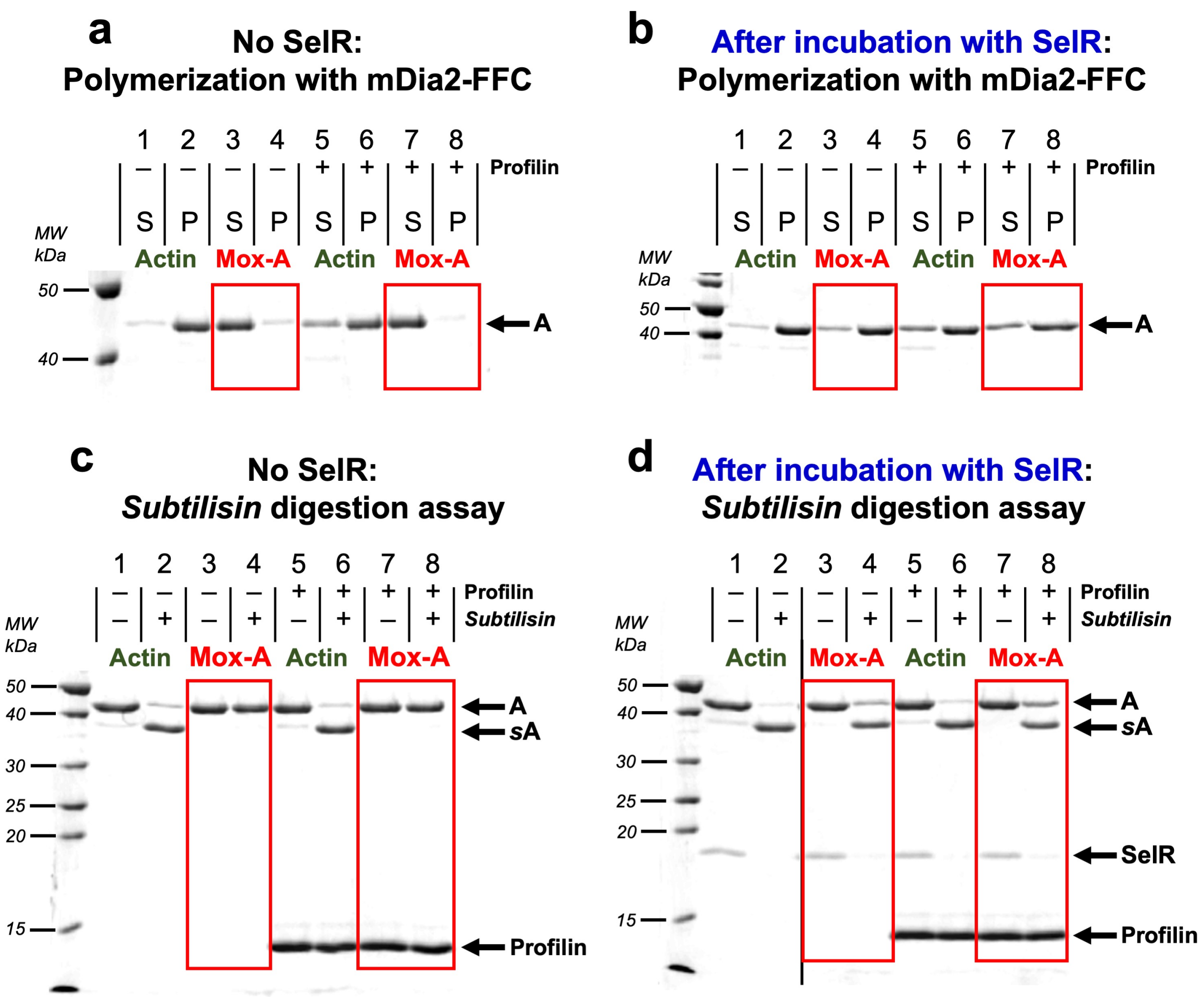

Supplementary Figure 1. SelR reduces Mox-actin in the presence of profilin and reverses its inhibition of profilin-assisted mDia2-FFC-mediated actin polymerization. (a-b) SelR reverses Mox-actin's effect on polymerization by profilin/mDia2-FFC. The samples of Mox- (Mox-A) and unoxidized Ca-ATP-actins with and without profilin were incubated with G-buffer (a) or SelR (b) for 100min at 20°C. After the incubation and Ca^{2+}/Mg^{2+} exchange, the samples were supplemented with polymerizing buffer and mDia2-FFC formin construct (30nM) and after 2-hour polymerization (20°C) they were subjected to the high-speed pelleting. Note, that after the incubation with SelR, the unoxidized and Mox-actin samples now show similar amounts of actin (A) in the pellets. This is consistent with the effective reduction of Mox-actin by SelR under our experimental conditions and its conversion to unoxidized actin with and without profilin present. S=soluble fraction. P=pellet fraction. [Actin]=2.25µM, [profilin]=6.75µM, [SelR]=0.57µM. n=2 separate experiments with similar results for a-b. **(c-d)** Confirmation of SelR's reduction of Mox-actin in the presence of profilin. Subtilisin digestion assays were employed to differentiate between actin and Mox-actin as previously described [1]. **(c)** Monomeric Ca-ATP-Mox-actin (Mox-A) is not digested by subtilisin regardless of profilin present (lanes 4 and 8, A). At the same time, unoxidized actin undergoes limited proteolysis under the same conditions (lanes 2 and 6, *s*A). Importantly, the presence of profilin does not affect the digestion pattern of unoxidized actin (compare lanes 2 and 6 in (c)), which validates the use of the subtilisin digestion assay in the presence of profilin. **(d)** After incubation with SelR, Mox-actin (Mox-A) shows a subtilisin digestion pattern similar to that of unoxidized actin. Thus, limited proteolysis confirms the results of the pelleting experiments and shows that Mox-actin is being reduced by SelR in the absence and presence of profilin. [Actin]=2.5µM, [profilin]=7.5µM, [SelR]=0.63µM. n=2 separate experiments with similar results for c-d. Short vertical black bar between lanes 2 and 3 indicates that they are not adjacent in the gel. Source data/uncropped gels for Suppl. Fig. 1 are provided as a Source Data file.

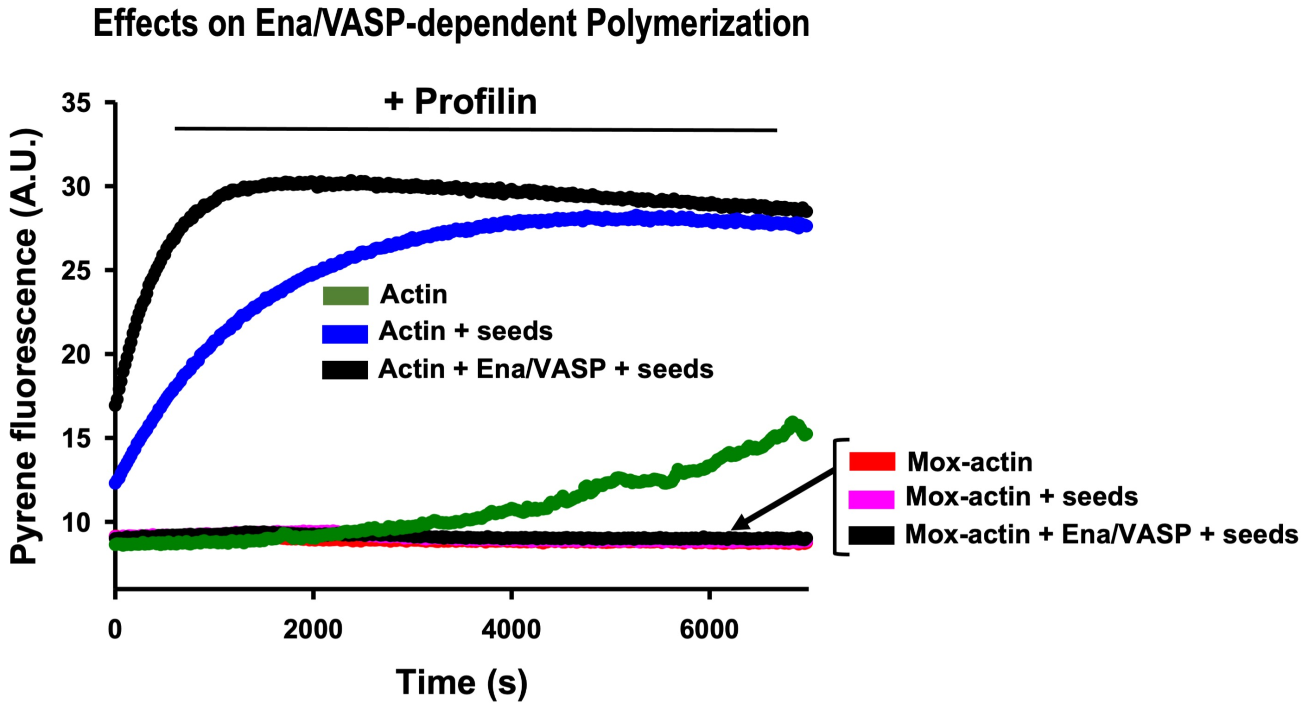

Supplementary Figure 2. Ena/VASP does not support Mox-actin assembly in the presence of profilin. [Actin]=3µM; [profilin]=9µM; [Ena/VASP]=25nM (in tetramers); [F-actin-phalloidin seeds]=0.25µM (stabilized with phalloidin (Ph) at 1:50 Ph:actin molar ratio). n=2 replicates with similar results. A.U. = arbitrary units. Source data are provided as a Source Data file.

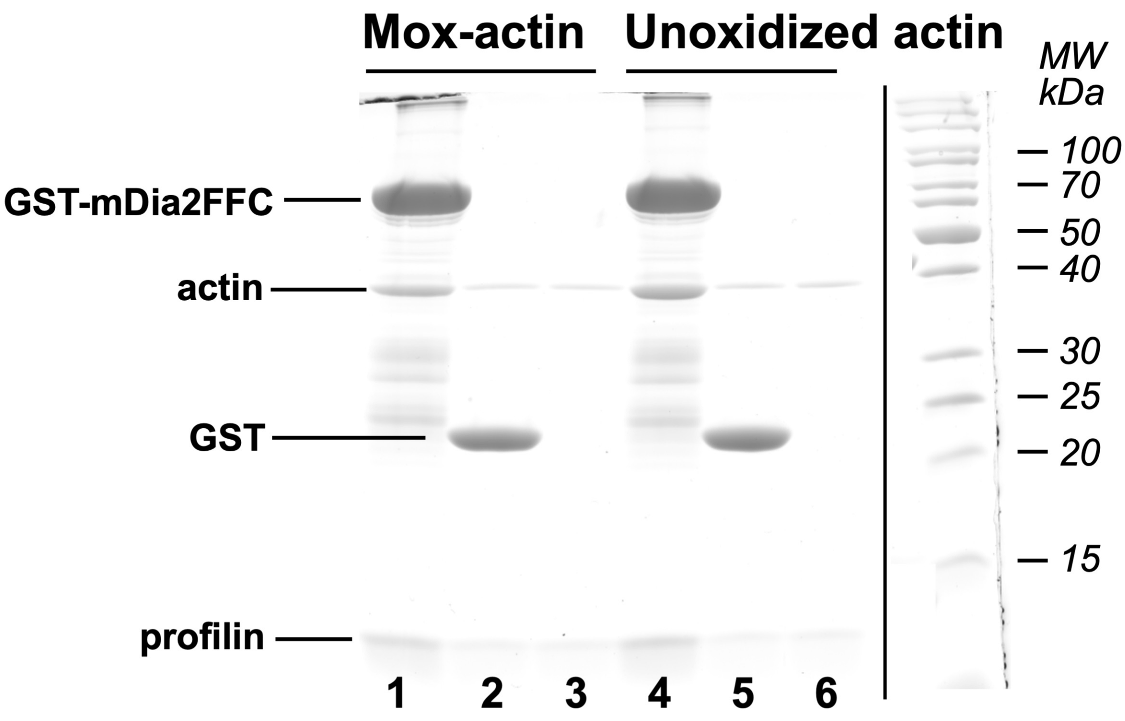

Supplementary Figure 3. Complexes of unoxidized and Mical-oxidized actin with profilin bind to mDia2-FF. Elution fractions were analyzed by SDS-PAGE. Lanes 1 and 4 – eluates from the GST-mDia2-FF-bound beads indicate that actin:profilin (lane 4) and Mox-actin:profilin complexes (lane 1) bind to formin. Controls: lanes 2 and 5 – show the extent of non-specific binding of actins and profilin to GST-bound beads; lanes 3 and 6 – show the extent of non-specific binding to the empty beads. [GST-mDia2-FF dimer]=0.5μM; [actin]=5μM; [profilin]=15μM. n=3 separate pull-down experiments with similar results. Vertical black line denotes that this figure is composed of two panels originating from the same gel (lines 1-6, left) and molecular weight ladder (right). Source data/uncropped gels are provided as a Source Data file.

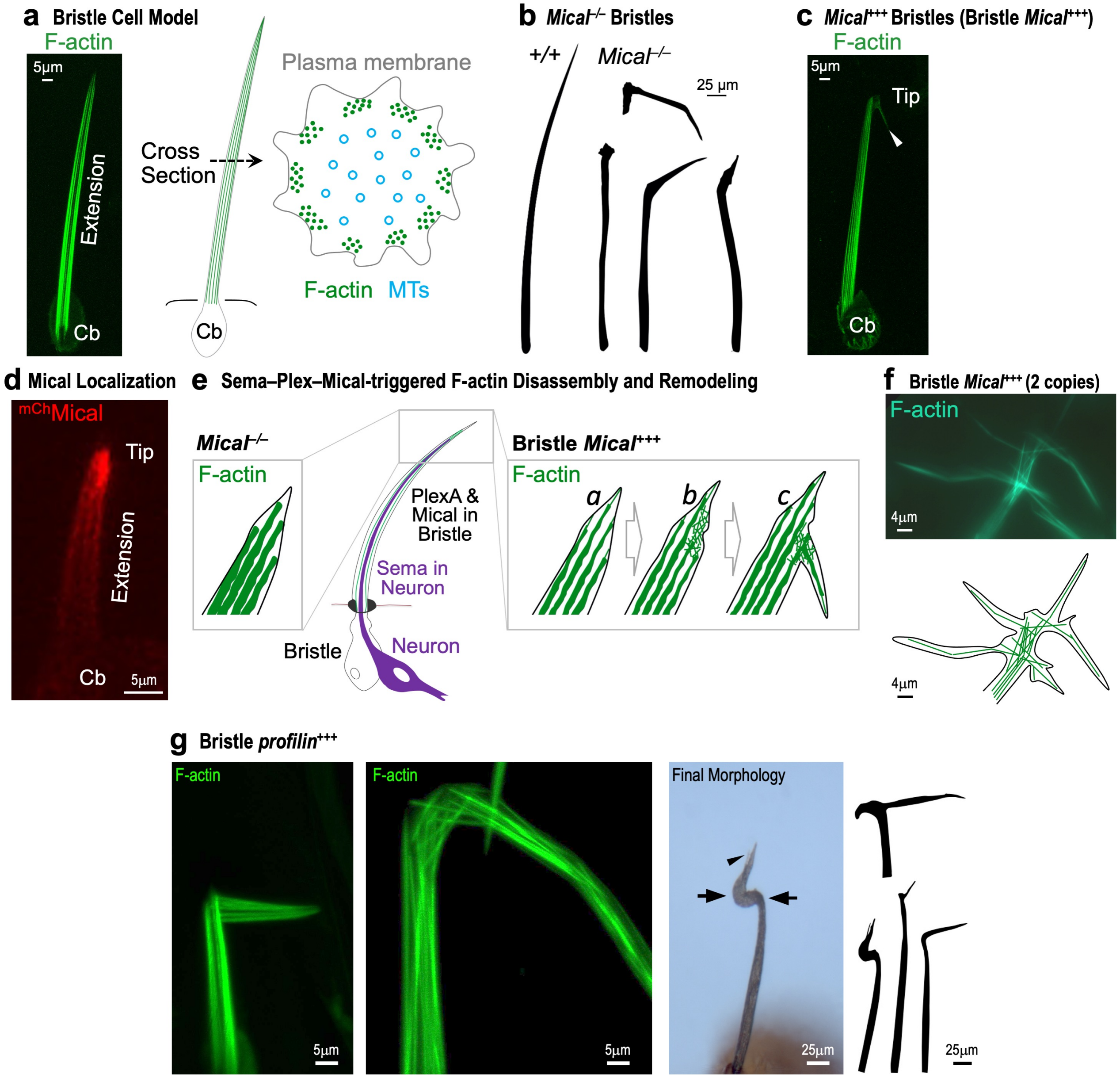

Supplementary Figure 4. The *Drosophila* single cell bristle system – and roles for Mical and profilin in directing bristle shape and F-actin organization. (**a**) Summary of the model bristle system. **(Left (Image))** Each bristle cell (*wild-type* as in **Fig. 4a**) extends a single F-actin (green) and microtubule-filled (not shown) membranous extension. Cb, cell body. **(Right (Diagram))** Cytoskeletal organization of the bristle cell model (wild-type as in (**a**), and **Fig. 4a**). Actin in bristles, as in neurons and other types of cells, is assembled into different structures (including branched/less bundled (lamellopodial-like) structures and bundled/less branched (filopodial-like) structures) and there is an interplay between these different organizational types of actin (e.g., see [2, 3, 4, 5, 6]). For example, as the bristle elongates, actin is polymerized at the tip and there is a predominance of dynamic actin filaments that are branched and not crosslinked (termed actin patches/snarls). When stabilized by crosslinking proteins and interactions with the plasma membrane, these actin patches function to shape, position, and become incorporated into F-actin bundles, which help to elongate and stabilize the bristle (depicted with green lines on left image and as dots in cross section (right diagram)). See [2, 3, 4, 7, 8, 9, 10, 11] for more details. (**b**) *Wild-type* (+/+) animals have slightly curved, unbranched bristles (see drawing, **(a)**, and **Fig. 4a**). Bristles in *Mical* "knockout" mutants (*Mical*$^{-/-}$) are straight and/or bent (i.e., stiffer, less curved) – and also show notable tip alterations (thick, non-tapered tips). These defects are the result of too much F-actin within the bristles of *Mical*$^{-/-}$ mutants (see (**e**) and [8, 12, 13] for more details). The presentation of these bristle drawings is modified from [12, 13].

(**c**) Elevating Mical levels within bristles (*Mical*$^{+++}$ Bristles (called Bristle *Mical*$^{+++}$ herein)) differs from the effects of *Mical*$^{-/-}$ and results in F-actin disassembly, which occurs not along the extent of the bristle but specifically at the bristle tip (which is where Mical localizes; see d), and then this F-actin disassembly is followed by subsequent F-actin assembly in a new direction (arrowhead, see also (**d-e**) and **Fig. 4b** for more details). (**d-f**) Previous results showing Mical's localization and effects on bristle F-actin disassembly and bristle remodeling. (**d**) As previously described (see [1, 8, 10, 11, 12, 13, 14, 15] for more details), both endogenous and overexpressed Mical localizes to the tip of the bristle during its extension. mCh (mCherry). Genotype: *UAS:*^{mCh}*Mical/+, B11-GAL4/+*. (**e**) Sema–Plex–Mical-triggered F-actin disassembly and remodeling. (**e**, **Left portion of Diagram**) Previous analyses (see [8, 10, 12] for more details) find that loss of Mical (*Mical*$^{-/-}$) results in too much F-actin/thicker bundles of F-actin (green) in bristles. (**e, Middle portion of Diagram**) Previous analyses (see [8, 10, 12] for more details), shows that Mical's activating receptor, Plexin (PlexA), is expressed in the bristle, and the Sema ligands for PlexA (Sema-1a and Sema-1b) are expressed in the neuron and its dendrite (purple) that extends alongside the developing bristle. Previous analyses also support a model that Sema activates PlexA/Mical signaling in the bristle to alter the F-actin cytoskeleton and shape of bristles (see [8, 10, 12] for more details). (**e, Right portion of Diagram**) Previous analyses (see [1, 8, 10, 11, 12, 13, 14, 15] for more details) find that Mical-dependent branching observed in **Fig. 4b** (Bristle *Mical*$^{+++}$) is the result of Mical's Redox-dependent F-actin (green) disassembly at a specific locale (b) – and then the subsequent remodeling and new assembly of the F-actin cytoskeleton to generate an F-actin-rich branch at that spot (c). Diagram adapted from [10]. (**f**) Previous results (see [8, 10, 12] for more details) find that further increasing the levels of Mical in the bristle (expressing 2 copies of the Mical transgene in the bristle (Bristle Mical^{+++} (2 copies)) results in additional F-actin disassembly and F-actin-rich branches. Modified from [12]. (**g**) Profilin (chickadee) also regulates the shape and F-actin organization of bristles [3, 16, 17]. In contrast to some lines expressing profilin (chickadee) in bristles, which enhance Mical's effects but exhibit no observable bristle defects on their own (see **Fig. 4d, f**), expression of some profilin (chickadee) lines on their own (*UAS:profilin/+, B11-GAL4/+*) or expression of higher levels of other *profilin* lines on their own (*UAS:profilin/+, UAS:profilin/+, B11-GAL4/+*) generates bristle defects including branches, bends, and bend-like branches. In particular, unlike the slightly curved, unbranched bristles of wild-type flies (**Fig. 4a**), elevating *profilin* levels specifically in bristles results in bristles that are bent (e.g., arrows and drawings), are split at their ends (e.g., drawings), and/or have slender barbs at the tip (e.g., arrowhead and drawings) (see also [17]). F-actin (green) is more numerous in bristles that have elevated levels of profilin and underlie these changes in bristle shape [17]. Note that based on these bends, tip alterations, and the presence of excess F-actin, elevating the bristle levels of profilin has similarities to *Mical*$^{-/-}$ mutant bristles (compare with b; [12, 17]). Yet, based on the small barbs/slight branches seen when profilin is overexpressed in bristles (and has also been previously described; [17]), we also noted some similarities to Mical overexpression bristles (Bristle *Mical*$^{+++}$*;* see **Fig. 4b**, and above in (**e-f**)). Genotype for the Neuronal *profilin*$^{+++}$ images in g: *UAS:profilin (chic78.3)/+, B11-GAL4/+.* The data which provides the basis for the summary images and drawings in (a-f) have been repeated multiple times and previously published (e.g., [2, 3, 4, 7, 8, 9, 10, 11]). For the experiments in (g), at least 2 independent experiments were performed with similar results (and these types of effects have also been previously described; [17]).

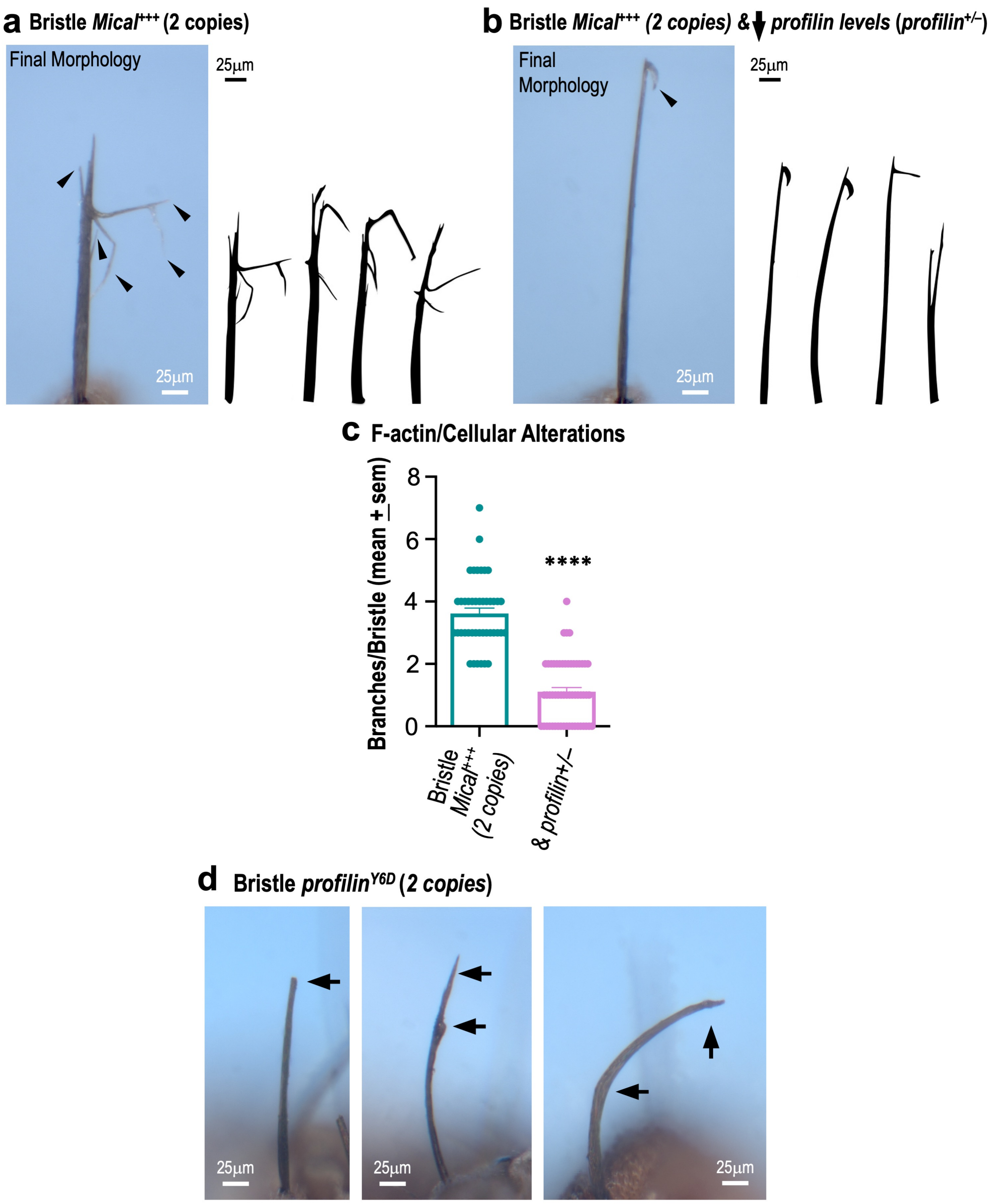

Supplementary Figure 5. Further analyses of Mical and profilin functionally interacting *in vivo* to mediate cellular remodeling. Note that for all experiments in (a-c), one copy of the bristle-specific GAL4 driver, *B11-GAL4*, and two copies of the Mical transgene, *UAS:Mical*, are used (and for ease of understanding, we call this genotype Bristle *Mical*$^{+++}$ (2 copies)). **(a)** Increasing *Mical* in bristle cells to higher levels (Bristle *Mical*$^{+++}$ (2 copies) results in more cellular remodeling and generates additional branches (black arrowheads and drawings). Genotype: *UAS:Mical/+, UAS:Mical/+, B11-GAL4/+* (n=42 cells assessed across 21 animals). (**b**) Decreasing the level of *profilin* (*profilin* heterozygous mutant (*profilin*$^{+/-}$)) in this Bristle *Mical*$^{+++}$ (2 copies) background, decreases Mical-mediated cellular remodeling (black arrowhead and drawings). Genotype: *UAS:Mical/+, UAS:Mical/+, B11-GAL4/+*, *chic*^{1320}/+ (n=56 cells assessed across 28 animals). (**c**) Quantification of the number of branches per bristle from the data in a and b. Means$\pm$SEM. ****p<0.0001; unpaired *t* test (two-tailed). Source data are provided as a Source Data file. **(d)** Overexpression of high levels (2 copies) of *profilin*^{Y6D} in bristles – a mutation known to induce a lower affinity of profilin for the polyproline tracks of actin elongation-promoting factors [18] – gives rise to severely stunted (about ½ the size of normal bristles) and misshapen bristles (arrows, and compare to **Fig. 4a**) that resemble the effects seen in *profilin*$^{-/-}$ mutants [3, 16, 17] and actin elongation-promoting factor knockdown mutants such as the formin *Dia* [19], where F-actin cannot assemble normally and so bristle elongation stalls. Genotype: *UAS:profilin*^{Y6D} *(4F)/+, UAS:profilin*^{Y6D} *(5F)/+, B11-GAL4/+*. At least 2 independent experiments were performed with similar results for each of the experiments in (a-d).

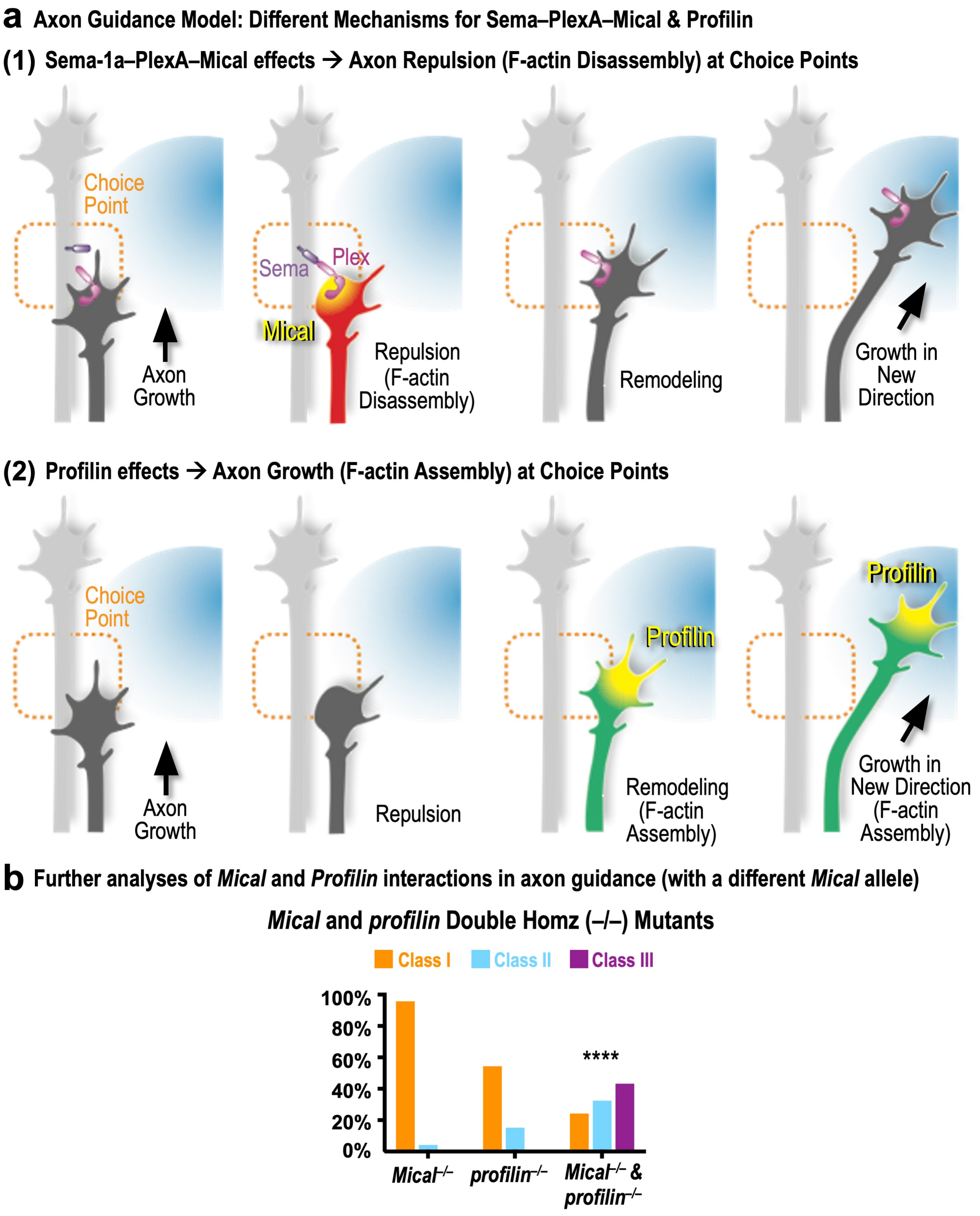

Supplementary Figure 6. Previous results find that *Sema–PlexA–Mical* mutants and *profilin* mutants generate similar axon guidance defects but use different mechanisms to guide axons. (**a**) Despite generating similar mutant axon guidance defects *in vivo* (see **Fig. 5a**), previous experimental results indicate that the mechanisms for how Sema-1a–PlexA–Mical and profilin exert their effects on the guidance of axons are different. (1) The extracellular transmembrane ligand Sema-1a (purple) serves as a repulsive guidance cue for its receptor PlexA (pink). Sema-1a/PlexA induce repulsion (defasciculation) of axons at choice points, so that axons grow away from the choice point in a new trajectory (model based on results in [20, 21, 22]). Likewise, Mical (yellow), which is an F-actin disassembly enzyme (reviewed in [23, 24, 25, 26]), interacts with the cytoplasmic region of PlexA, and is important for Sema-1a–PlexA repulsion/defasciculation at choice points (model based on results in [12, 27]). (2) Profilin, is also required for axons to grow away from choice points, but works in a different manner. In particular, previous results support that profilin works at least in part by assisting actin assembly (reviewed in [28, 29]), and coupled with observations that axon outgrowth and filopodial length is reduced in *Drosophila profilin*$^{-/-}$ mutants [30, 31], results indicate that profilin (yellow) is important for driving actin assembly that is needed for axons to move away from choice points (e.g., model based on results from [30, 31, 32, 33, 34]). Diagrams adapted from [35]. (**b**) Further analysis of interactions between *Mical* mutants and *profilin* mutants. Similar to Fig. 5e: analyses of *Mical*$^{-/-}$ and *profilin*$^{-/-}$ double homozygous (Homz) mutants using a different *Mical*$^{-/-}$ allele in combination with

profilin$^{-/-}$ further reveals that *Mical*$^{-/-}$ and *profilin*$^{-/-}$ double Homz mutants exhibit significantly more severe guidance defects than either homozygous mutant alone – including Class III defects, in which axons are excessively/repetitively bundled together (arrowheads). Scoring system adapted from [31]. Defects: Class I (affecting longitudinal bundle 3), Class II (affecting longitudinal bundles 2 and 3), Class III (affecting longitudinal bundles 1, 2, and 3). *Mical*$^{-/-}$=*Mical*^{K1496}/*Mical*^{K1496} (n=23 animals), *profilin*$^{-/-}$=*chic*^{221}/*chic*^{221} (n=46 animals; see also [31, 33, 34]), *Mical*$^{-/-}$ & *profilin*$^{-/-}$=*Mical*^{K1496}/*Mical*^{K1496} & *chic*^{221}/*chic*^{221} (n=37 animals). ****p<0.0001; X^2 test. $\geq$2 independent experiments gave similar results. Source data are provided as a Source Data file.

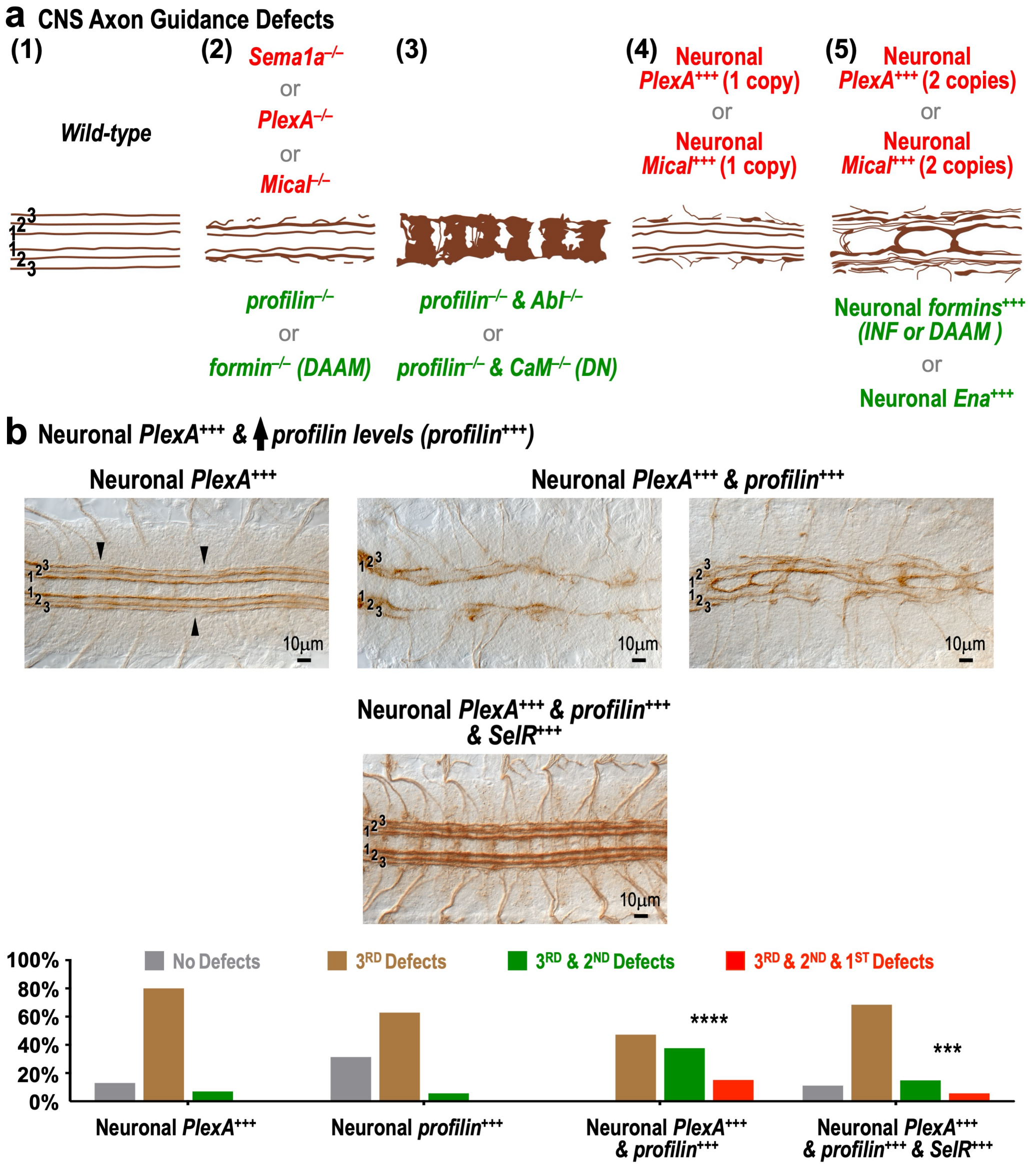

Supplementary Figure 7. The similarity of the effects of Sema–Plex–Mical and profilin/actin elongation-promoting factors on the guidance of CNS axons, and further analysis of Sema–Plex–Mical and profilin's interactions to direct axon guidance. (a) Sema–Plex–Mical repulsive factors and profilin/actin elongation-promoting factors exhibit similar effects on CNS axon guidance. **(1)** In wild-type embryos, three (1, 2, 3) evenly spaced and uniformly thick longitudinal axon bundles are detected on each side of the CNS using the 1D4 antibody to neural cell adhesion molecule (NCAM)/fasciclin II [32]. **(2)** Loss of *Sema1a* (*Sema1a$^{-/-}$*), *PlexA* (*PlexA$^{-/-}$*), *or Mical* (*Mical$^{-/-}$*) affects the guidance of CNS longitudinal axons. In particular, axons become too tightly bundled together and the 3rd longitudinal is seen to be missing and/or "fused" with the 2nd longitudinal [12, 20, 21]. Interesting, loss of *profilin* (*profilin$^{-/-}$*) also affects CNS axon guidance and gives rise to missing 3rd longitudinals [31, 33, 34]. These types of missing 3rd longitudinal CNS axon guidance defects are also seen in mutations of the formin DAAM (*DAAM$^{-/-}$*) [34]. *Ena/VASP* (*ena$^{-/-}$*) mutants also have CNS axon guidance defects [36, 37, 38]. Drawing based on [20, 21, 34]. **(3)** The *profilin* mutant (*profilin$^{-/-}$*) CNS axon guidance defects become more severe when embryos are both homozygous for mutations in *profilin* (*profilin$^{-/-}$*) and heterozygous or homozygous for mutations of profilin-interacting proteins such as *Abl*, the formin *DAAM,* or *calmodulin (CaM)* (i.e., double mutants; [31, 33, 34]). In particular, axons become severely bundled together (too much fasciculation) within each of the different segments of the embryo, such that the axons abnormally cross the midline and have a decreased ability to change their trajectory and project down the cord. Drawing based on [31, 33]. The interaction between profilin and the formin DAAM was shown with another axonal marker (BP102) [34] and so is not illustrated here.

Note also that profilin, which is also supplied by the mother (i.e., maternally), has not been removed in these *profilin*$^{-/-}$ mutants ((2)-(3); [31, 33]; i.e., they are the typical zygotic mutants like the other mutants illustrated here in (2-3)) and so that is why earlier developmental events are not affected. Likewise, because this maternally-supplied profilin is not removed, double mutant combinations between *profilin*$^{-/-}$ and its interactors result in more severe defects than either homozygous mutant alone [31, 33, 34] (and that is why double mutant combinations have been used to look for interactions between profilin and other proteins) [31, 33, 34]. (**4-5**) Neuronal overexpression of *PlexA* and/or *Mical* also gives rise to CNS axon guidance defects that resemble but are distinctively different than *Sema1a* (*Sema1a*$^{-/-}$*), PlexA* (*PlexA*$^{-/-}$*),* or *Mical* (*Mical*$^{-/-}$*)* mutants [12, 21, 35, 39] – and they are similar to neuronal overexpression of profilin-interacting actin elongation-promoting factors such as the formin DAAM, the formin INF (formin3), and Ena/VASP [34, 40, 41]. (**4**) Defects in the guidance of CNS axons, like the defects in the bristle model (compare **Fig. 4b, f** and **Supplementary Fig. 5a, c**), become more severe as the levels of *Mical* or *PlexA* are increased. In particular, neuronal overexpression of low levels of *PlexA*$^{+++}$ (1 copy) [15, 21, 35] or neuronal overexpression of low levels of *Mical*$^{+++}$ (1 copy) [12] result predominantly in disorganization of the outermost (3rd) longitudinal with axons less tightly fasciculated and projecting away from the CNS (defects that are consistent with increased/too-much repulsion of axons). Drawing based on [21]. (**5**) When overexpressing high levels of PlexA or Mical in neurons (such as 2 copies of *PlexA* or 2 copies of *Mical* or 1 copy of *PlexA* and 1 copy of *Mical*) more severe CNS axon guidance defects occur [12, 14, 35, 39]. In particular, whereas in loss of Sema–Plexin–Mical repulsion (*Sema1a* (*Sema1a*$^{-/-}$*), PlexA* (*PlexA*$^{-/-}$*), or Mical* (*Mical*$^{-/-}$) mutants), axons become too tightly bundled/fasciculated together [12, 20, 21, 35, 39], in neuronal overexpression of *PlexA* (*PlexA*$^{+++}$) *or Mical* (*Mical*$^{+++}$) (i.e., increasing Sema–Plexin–Mical repulsion) each of the three longitudinals become less bundled/more defasciculated, and project out of the CNS and/or across the midline [12, 21, 35, 39]. These types of CNS axon guidance defects are also seen following increasing the neuronal levels of the *formin DAAM* (constituitively active (CA) form of DAAM; [34]), the *formin INF* (*formin3*) [40], or *Ena/VASP* [41]. Drawing based on [12, 34, 35, 39]. (**b**) Increasing the neuronal levels of *profilin* increases PlexA-mediated alterations to axon guidance, and these effects can be rescued by increasing the neuronal levels of SelR. Scoring of defects similar to Fig. 6e. **Neuronal *PlexA*$^{+++}$:** Increasing the neuronal levels of *PlexA* results in CNS axon guidance defects, with defects typically resulting in the diminished presence of the 3rd longitudinal (arrowheads). These results are similar to that described previously for low level neuronal overexpression of PlexA [15, 21, 35] and see also (a (4)). **Neuronal *PlexA*$^{+++}$ & *profilin*$^{+++}$:** Neuronal overexpression of *PlexA* in combination with increasing the neuronal levels of *profilin* results in a significant increase in the severity of guidance defects with defects now affecting all three longitudinals (image and graph). Note that the axons are abnormally separated from one another (i.e., not excessively bundled together) and are present in thinner bundles so that it appears that there is a paucity of axons. These defects are similar to what has been described previously for high levels of neuronal overexpression of PlexA [14, 35, 39] (and see also above in (a (5)). They are also similar to what has been previously observed with low levels of PlexA + other Plexin interacting proteins [1, 15, 35, 42]. Compare also with **Fig. 6e**. **Neuronal *PlexA*$^{+++}$ & *profilin*$^{+++}$ & *SelR*$^{+++}$:** Increasing the neuronal levels of *SelR* significantly suppresses the effects seen when increasing the neuronal levels of *PlexA* and *profilin*, such that a more normal pattern of axons/bundled axons is observed in all three longitudinals. The effects of increasing the neuronal levels of profilin on its own (**Neuronal *profilin*$^{+++}$)** can be seen in Fig. 6a and in the graph. Neuronal *PlexA*$^{+++}$*=UAS:PlexA/+, ELAV-GAL4/+* (n=100 animals; see also [15, 21, 35]*),* Neuronal *profilin*$^{+++}$*=UAS:profilin /+, ELAV-GAL4/+* (n=35 animals). Neuronal *PlexA*$^{+++}$ *& profilin*$^{+++}$*: UAS:PlexA/+, UAS:profilin /+, ELAV-GAL4/+* (n=53 animals). Neuronal *PlexA*$^{+++}$ *& profilin*$^{+++}$ *& SelR*$^{+++}$*=UAS:PlexA/+, UAS:profilin /+, UAS:SelR/+, ELAV-GAL4/+* (n=54 animals). ****p<0.0001; X^2 test for comparing the data in the green and red bars in the first three genotypes on the graph, and ***p=0.0005; X^2 test for comparing the data in the green and red bars in the 3rd and 4th genotypes on the graph. At least 2 independent experiments were performed with similar results for each of the experiments in (b). Source data are provided as a Source Data file.

Transgene	Primers (5' to 3')	
Drosophila Profilin (chickadee)	Forward	AGCTGAATTCATGAGCTGGCAAGATTATGT
	Reverse	AGCTTCTAGACTAGTACCCGCAAGTAATCA
Drosophila Profilin (chickadee) ^{Y6D}	Forward	AGCTGAATTCATGAGCTGGCAAGATTATGT
	Reverse	AGCTTCTAGACTAGTACCCGCAAGTAATCA

Supplementary Table 1. Primers

Supplementary References

1. Grintsevich EE, Yesilyurt HG, Rich SK, Hung RJ, Terman JR, Reisler E. F-actin dismantling through a redox-driven synergy between Mical and cofilin. *Nat Cell Biol* **18**, 876-885 (2016).

2. Tilney LG, DeRosier DJ. How to make a curved Drosophila bristle using straight actin bundles. *Proc Natl Acad Sci U S A* **102**, 18785-18792 (2005).

3. Frank DJ, Hopmann R, Lenartowska M, Miller KG. Capping protein and the Arp2/3 complex regulate nonbundle actin filament assembly to indirectly control actin bundle positioning during Drosophila melanogaster bristle development. *Mol Biol Cell* **17**, 3930-3939 (2006).

4. Wu J, Wang H, Guo X, Chen J. Cofilin-mediated actin dynamics promotes actin bundle formation during Drosophila bristle development. *Mol Biol Cell* **27**, 2554-2564 (2016).

5. Chhabra ES, Higgs HN. The many faces of actin: matching assembly factors with cellular structures. *Nat Cell Biol* **9**, 1110-1121 (2007).

6. Dent EW, Gupton SL, Gertler FB. The growth cone cytoskeleton in axon outgrowth and guidance. *Cold Spring Harb Perspect Biol* **3**, a001800 (2011).

7. Sutherland JD, Witke W. Molecular genetic approaches to understanding the actin cytoskeleton. *Curr Opin Cell Biol* **11**, 142-151 (1999).

8. Hung R-J, Terman JR. Extracellular inhibitors, repellents, and Semaphorin/Plexin/MICAL-mediated actin filament disassembly. *Cytoskeleton* **68**, 415-433 (2011).

9. Bitan A, Guild G, Abdu U. The highly elongated Drosophila mechanosensory bristle--a new model for studying polarized microtubule function. *Fly* **4**, 246-248 (2010).

10. Rich SK, Terman JR. Axon formation, extension, and navigation: only a neuroscience phenomenon? *Curr Opin Neurobiol* **53**, 174-182 (2018).

11. Rich SK, Baskar R, Terman JR. Propagation of F-actin disassembly via Myosin15-Mical interactions. *Sci Adv* **7**, (2021).

12. Hung RJ*, et al.* Mical links semaphorins to F-actin disassembly. *Nature* **463**, 823-827 (2010).

13. Hung RJ, Pak CW, Terman JR. Direct redox regulation of F-actin assembly and disassembly by Mical. *Science* **334**, 1710-1713 (2011).

14. Hung RJ, Spaeth CS, Yesilyurt HG, Terman JR. SelR reverses Mical-mediated oxidation of actin to regulate F-actin dynamics. *Nat Cell Biol* **15**, 1445-1454 (2013).

15. Yoon J, Kim SB, Ahmed G, Shay JW, Terman JR. Amplification of F-Actin Disassembly and Cellular Repulsion by Growth Factor Signaling. *Dev Cell* **42**, 117-129 (2017).

16. Verheyen EM, Cooley L. Profilin mutations disrupt multiple actin-dependent processes during Drosophila development. *Development* **120**, 717-728 (1994).

17. Hopmann R, Miller KG. A balance of capping protein and profilin functions is required to regulate actin polymerization in Drosophila bristle. *Mol Biol Cell* **14**, 118-128 (2003).

18. Kovar DR, Harris ES, Mahaffy R, Higgs HN, Pollard TD. Control of the assembly of ATP- and ADP-actin by formins and profilin. *Cell* **124**, 423-435 (2006).

19. Lu Q, Adler PN. The diaphanous gene of Drosophila interacts antagonistically with multiple wing hairs and plays a key role in wing hair morphogenesis. *PLoS One* **10**, e0115623 (2015).

20. Yu HH, Araj HH, Ralls SA, Kolodkin AL. The transmembrane Semaphorin Sema I is required in Drosophila for embryonic motor and CNS axon guidance. *Neuron* **20**, 207-220. (1998).

21. Winberg ML*, et al.* Plexin A is a neuronal semaphorin receptor that controls axon guidance. *Cell* **95**, 903-916 (1998).

22. Yu HH, Huang AS, Kolodkin AL. Semaphorin-1a acts in concert with the cell adhesion molecules fasciclin II and connectin to regulate axon fasciculation in Drosophila. *Genetics* **156**, 723-731 (2000).

23. Alto LT, Terman JR. MICALs. *Curr Biol* **28**, R538-R541 (2018).

24. Manta B, Gladyshev VN. Regulated methionine oxidation by monooxygenases. *Free Radic Biol Med* **109**, 141-155 (2017).

25. Vanoni MA. Structure-function studies of MICAL, the unusual multidomain flavoenzyme involved in actin cytoskeleton dynamics. *Arch Biochem Biophys* **632**, 118-141 (2017).

26. Fremont S, Romet-Lemonne G, Houdusse A, Echard A. Emerging roles of MICAL family proteins - from actin oxidation to membrane trafficking during cytokinesis. *J Cell Sci* **130**, 1509-1517 (2017).

27. Terman JR, Mao T, Pasterkamp RJ, Yu HH, Kolodkin AL. MICALs, a family of conserved flavoprotein oxidoreductases, function in plexin-mediated axonal repulsion. *Cell* **109**, 887-900 (2002).

28. Courtemanche N. Mechanisms of formin-mediated actin assembly and dynamics. *Biophys Rev* **10**, 1553-1569 (2018).

29. Chesarone MA, DuPage AG, Goode BL. Unleashing formins to remodel the actin and microtubule cytoskeletons. *Nat Rev Mol Cell Biol* **11**, 62-74 (2010).

30. Goncalves-Pimentel C, Gombos R, Mihaly J, Sanchez-Soriano N, Prokop A. Dissecting regulatory networks of filopodia formation in a Drosophila growth cone model. *PLoS One* **6**, e18340 (2011).

31. Wills Z, Marr L, Zinn K, Goodman CS, Van Vactor D. Profilin and the Abl tyrosine kinase are required for motor axon outgrowth in the Drosophila embryo. *Neuron* **22**, 291-299 (1999).

32. Van Vactor D, Sink H, Fambrough D, Tsoo R, Goodman CS. Genes that control neuromuscular specificity in Drosophila. *Cell* **73**, 1137-1153 (1993).

33. Kim YS, Furman S, Sink H, VanBerkum MF. Calmodulin and profilin coregulate axon outgrowth in Drosophila. *J Neurobiol* **47**, 26-38 (2001).

34. Matusek T*, et al.* Formin proteins of the DAAM subfamily play a role during axon growth. *J Neurosci* **28**, 13310-13319 (2008).

35. Yang T, Terman JR. 14-3-3epsilon Couples Protein Kinase A to Semaphorin Signaling and Silences Plexin RasGAP-Mediated Axonal Repulsion. *Neuron* **74**, 108-121 (2012).

36. Gertler FB*, et al.* enabled, a dosage-sensitive suppressor of mutations in the Drosophila Abl tyrosine kinase, encodes an Abl substrate with SH3 domain-binding properties. *Genes Dev* **9**, 521-533 (1995).

37. Wills Z, Bateman J, Korey CA, Comer A, Van Vactor D. The tyrosine kinase Abl and its substrate enabled collaborate with the receptor phosphatase Dlar to control motor axon guidance. *Neuron* **22**, 301-312 (1999).

38. Bashaw GJ, Kidd T, Murray D, Pawson T, Goodman CS. Repulsive axon guidance: Abelson and Enabled play opposing roles downstream of the roundabout receptor. *Cell* **101**, 703-715. (2000).

39. Ayoob JC, Yu HH, Terman JR, Kolodkin AL. The Drosophila receptor guanylyl cyclase Gyc76C is required for semaphorin-1a-plexin A-mediated axonal repulsion. *J Neurosci* **24**, 6639-6649 (2004).

40. Tanaka H, Takasu E, Aigaki T, Kato K, Hayashi S, Nose A. Formin3 is required for assembly of the F-actin structure that mediates tracheal fusion in Drosophila. *Dev Biol* **274**, 413-425 (2004).

41. Cheong HSJ, Nona M, Guerra SB, VanBerkum MF. The first quarter of the C-terminal domain of Abelson regulates the WAVE regulatory complex and Enabled in axon guidance. *Neural Dev* **15**, 7 (2020).

42. Cho JY, Chak K, Andreone BJ, Wooley JR, Kolodkin AL. The extracellular matrix proteoglycan perlecan facilitates transmembrane semaphorin-mediated repulsive guidance. *Genes Dev* **26**, 2222-2235 (2012).
