# Supplementary material for: Profilin and Mical combine to impair F-actin assembly and promote disassembly and remodeling
Source: Nat Commun. 2021 Sep 20;12:5542. doi: 10.1038/s41467-021-25781-3 (PMC8452626; doi:10.1038/s41467-021-25781-3)
Supplement: Supplementary file 2 — Reporting Summary [file 41467_2021_25781_MOESM2_ESM.pdf]

nature research

Corresponding author(s): Elena E. Grintsevich, Emil Reisler, Jonathan R. Terman

Last updated by author(s): Jul 16, 2021

Reporting Summary

Nature Research wishes to improve the reproducibility of the work that we publish. This form provides structure for consistency and transparency in reporting. For further information on Nature Research policies, see Authors & Referees and the Editorial Policy Checklist .

Statistics

For all statistical analyses, confirm that the following items are present in the figure legend, table legend, main text, or Methods section.

n/a	Confirmed	
☐	☒	The exact sample size (*n*) for each experimental group/condition, given as a discrete number and unit of measurement
☐	☒	A statement on whether measurements were taken from distinct samples or whether the same sample was measured repeatedly
☐	☒	The statistical test(s) used AND whether they are one- or two-sided<br>*Only common tests should be described solely by name; describe more complex techniques in the Methods section.*
☒	☐	A description of all covariates tested
☒	☐	A description of any assumptions or corrections, such as tests of normality and adjustment for multiple comparisons
☐	☒	A full description of the statistical parameters including central tendency (e.g. means) or other basic estimates (e.g. regression coefficient) AND variation (e.g. standard deviation) or associated estimates of uncertainty (e.g. confidence intervals)
☐	☒	For null hypothesis testing, the test statistic (e.g. *F*, *t*, *r*) with confidence intervals, effect sizes, degrees of freedom and *P* value noted<br>*Give P values as exact values whenever suitable.*
☒	☐	For Bayesian analysis, information on the choice of priors and Markov chain Monte Carlo settings
☒	☐	For hierarchical and complex designs, identification of the appropriate level for tests and full reporting of outcomes
☒	☐	Estimates of effect sizes (e.g. Cohen's *d*, Pearson's *r*), indicating how they were calculated

Our web collection on statistics for biologists contains articles on many of the points above.

Software and code

Policy information about availability of computer code

Data collection	As described in the Methods of the paper: TIRF microscopy was performed with a DMI6000 TIRF microscope (Leica), Confocal microscopy imaging was performed with a Zeiss LSM710 and the aid of Zeiss Zen lite software (Ver #8.1); light microscopy was performed with a Zeiss Discovery M2 Bio stereomicroscope and Zeiss Axiovision software (Ver #4.8.2); and DIC microscopy imaging was performed with the aid of Zeiss Axioimager microscope and Zeiss Axiovision software (Ver #4.8.2).
Data analysis	As described in the Methods of the paper: Fiji (ImageJ, Ver #1.5.3a) software was used for gel/protein quantification and measurements on light microscopy images; presentation of light microscopy images and drawings was performed with the aid of Zeiss Axiovision software (Ver #4.8.2), Extended Focus software (Ver #1), GraphPad Prism software (Ver #8.4.3), SigmaPlot (Ver #11), Microsoft Powerpoint software (Ver #16.49).

For manuscripts utilizing custom algorithms or software that are central to the research but not yet described in published literature, software must be made available to editors/reviewers. We strongly encourage code deposition in a community repository (e.g. GitHub). See the Nature Research guidelines for submitting code & software for further information.

Data

Policy information about availability of data

All manuscripts must include a data availability statement. This statement should provide the following information, where applicable:

- Accession codes, unique identifiers, or web links for publicly available datasets
- A list of figures that have associated raw data
- A description of any restrictions on data availability

A "Data Availability" section is provided in the Methods and includes the following statement within it: "All data generated or analysed during this study are included in this published article and its Supplementary Information files."

Field-specific reporting

Please select the one below that is the best fit for your research. If you are not sure, read the appropriate sections before making your selection.

- [x] Life sciences
- [ ] Behavioural & social sciences
- [ ] Ecological, evolutionary & environmental sciences

For a reference copy of the document with all sections, see nature.com/documents/nr-reporting-summary-flat.pdf

Life sciences study design

All studies must disclose on these points even when the disclosure is negative.

Sample size

The sample size was chosen based on what is standardly done in the field in published manuscripts. We have referenced those published manuscripts in the methods and figure legends. For example, for Figure 4f, g and Suppl Fig. 5c, references are given on PP. 29-32. For Figures 5c, e and 6c, d, e, f and Suppl Figs. 6b and 7b, references are given on PP. 32-34. Statistical methods were not used to predetermine sample size. For the animal studies, differences between experimental and control conditions were highly notable with relatively little variability – and so the sample size was larger than what was needed to ensure adequate power to detect an effect. We have also indicated this information in the manuscript on PP. 34-35.

Data exclusions

Data were not excluded.

Replication

Main figures (and this info is in the Figure legends) and all showed similar results: 1c, n=2 independent experiments with different preps of both actins; 1d, n=2 separate experiments; 2a-d, n=2 separate experiments; 2e-f, n=2 separate experiments; 3a, n=3 separate experiments; 3b, n=2 samples, 6-7 random fields were imaged per condition in each experiment; 3c, n=3 separate experiments, 3d: n=3 separate experiments; 3e, n=2 separate experiments; 3f, n=2 separate experiments. At least 2 independent experiments were performed for the data displayed in Figs. 4-6 and this is stated in the figure legends.

Supplementary Figures (and this info is in the Supplementary Figure legends) and all showed similar results: Suppl. Fig. 1, n=2 separate experiments; Suppl. Fig. 2, n=2 replicates; Suppl Fig. 3, n=3 separate pull-down experiments. At least 2 independent experiments were performed for the data displayed in Suppl. Figs. 4-7 and this is stated in the Supplementary figure legends.

Randomization

Animal experiments were not randomized. Animals of the correct genotype were determined and those collected of that genotype were included as data. Likewise, for biochemical experiments, samples were grouped together based on experimental conditions and collected data points for those experiments are presented.

Blinding

In all genetic experiments, the genotype needed to be determined based on different fly genetic/chromosome markers, so blinding was not employed. Likewise, for the biochemical experiments, different proteins and reagents for the particular data set needed to be added and then analyzed using specific technical approaches and expertise, and so blinding was not employed. For both genetic and biochemical experiments, differences between the control and experimental conditions were highly notable and reproducible in both biological and technical replicates.

Reporting for specific materials, systems and methods

We require information from authors about some types of materials, experimental systems and methods used in many studies. Here, indicate whether each material, system or method listed is relevant to your study. If you are not sure if a list item applies to your research, read the appropriate section before selecting a response.

Materials & experimental systems

n/a	Involved in the study	
[ ]	[x]	Antibodies
[x]	[ ]	Eukaryotic cell lines
[x]	[ ]	Palaeontology
[ ]	[x]	Animals and other organisms
[x]	[ ]	Human research participants
[x]	[ ]	Clinical data

Methods

n/a	Involved in the study	
[x]	[ ]	ChIP-seq
[x]	[ ]	Flow cytometry
[x]	[ ]	MRI-based neuroimaging

Antibodies

Antibodies used

Monoclonal antibody to Drosophila N-CAM/Fasciclin II (1:4 dilution; Cat # Clone 1D4 anti-fasciclin II supernatant, purchased from Developmental Studies Hybridoma Bank). Antibody to GFP (1:1000; A-11122; purchased from ThermoFisher). HRP-conjugated anti-mouse IgG secondary antibody (1:500; 115-035-003, purchased from Jackson). HRP-conjugated anti-rabbit IgG secondary antibody (1:100; G-21234, purchased from ThermoFisher). Listed on PP. 32-34 of the Methods.

Validation

Clone 1D4 is a monoclonal antibody that labels Drosophila motor and specific central nervous system (CNS) axons and has been used extensively by multiple labs including us for many years as a marker to observe specific CNS and motor axons. A few of the many references using this antibody in this manner are provided on PP. 32-34 of the Methods (e.g., Van Vactor et al, Cell, 1993, Yu et al, Neuron, 1998, Terman et al, Cell, 2002). The antibody to GFP (A-11122; purchased from ThermoFisher) has also been

used extensively by multiple labs including us for many years to detect GFP-tagged proteins in tissue. We reference our previous work with the antibody on P. 34 of the Methods (Hung et al, Nature, 2010) and the ThermoFisher website lists many other studies employing this antibody.

Animals and other organisms

Policy information about studies involving animals; ARRIVE guidelines recommended for reporting animal research

Laboratory animals	Both males and females of the fly strain Drosophila melanogaster were used. Animals of the following ages were used for the study: embryos, pupae, and adults (PP. 29-34 of Methods).
Wild animals	No wild animals were used in the study
Field-collected samples	No field collected samples were used in the study
Ethics oversight	All animal work was done in accordance with University guidelines and the study did not require any additional ethical approval

Note that full information on the approval of the study protocol must also be provided in the manuscript.
